# Designing Oxide Catalysts for Oxygen Electrocatalysis: Insights from Mechanism to Application

**DOI:** 10.1007/s40820-023-01152-z

**Published:** 2023-07-29

**Authors:** Ning Han, Wei Zhang, Wei Guo, Hui Pan, Bo Jiang, Lingbao Xing, Hao Tian, Guoxiu Wang, Xuan Zhang, Jan Fransaer

**Affiliations:** 1 Department of Materials Engineering, KU Leuven, 3001 Leuven, Belgium; 2 ZJU-Hangzhou Global Scientific and Technological Innovation Centre, Zhejiang University, Hangzhou, 311200 People’s Republic of China; 3 Department of Physics and Astronomy, KU Leuven, 3001 Leuven, Belgium; 4 Key Laboratory of Ocean Energy Utilization and Energy Conservation of Ministry of Education, Dalian University of Technology, Dalian, 116023 People’s Republic of China; 5 School of Chemistry and Chemical Engineering, Shandong University of Technology, Zibo, 255000 People’s Republic of China; 6 Centre for Clean Energy Technology, Faculty of Science, University of Technology Sydney, Broadway, PO Box 123, Ultimo, NSW 2007 Australia

**Keywords:** Oxygen evolution, Oxygen reduction, Oxide catalysts, Catalyst design, Fuel cell, Metal–air batteries

## Abstract

The electrochemical oxygen reduction reaction (ORR) and oxygen evolution reaction (OER) are fundamental processes in a range of energy conversion devices such as fuel cells and metal–air batteries. ORR and OER both have significant activation barriers, which severely limit the overall performance of energy conversion devices that utilize ORR/OER. Meanwhile, ORR is another very important electrochemical reaction involving oxygen that has been widely investigated. ORR occurs in aqueous solutions via two pathways: the direct 4-electron reduction or 2-electron reduction pathways from O_2_ to water (H_2_O) or from O_2_ to hydrogen peroxide (H_2_O_2_). Noble metal electrocatalysts are often used to catalyze OER and ORR, despite the fact that noble metal electrocatalysts have certain intrinsic limitations, such as low storage. Thus, it is urgent to develop more active and stable low-cost electrocatalysts, especially for severe environments (e.g., acidic media). Theoretically, an ideal oxygen electrocatalyst should provide adequate binding to oxygen species. Transition metals not belonging to the platinum group metal-based oxides are a low-cost substance that could give a d orbital for oxygen species binding. As a result, transition metal oxides are regarded as a substitute for typical precious metal oxygen electrocatalysts. However, the development of oxide catalysts for oxygen reduction and oxygen evolution reactions still faces significant challenges, e.g., catalytic activity, stability, cost, and reaction mechanism. We discuss the fundamental principles underlying the design of oxide catalysts, including the influence of crystal structure, and electronic structure on their performance. We also discuss the challenges associated with developing oxide catalysts and the potential strategies to overcome these challenges.

## Highlights

- Fundamental principles underlying the design of oxide catalysts, including the influence of crystal structure, and electronic structure on their performance are summarized and analyzed.
- Challenges associated with developing oxide catalysts and the potential strategies are discussed.

## Graphical abstract

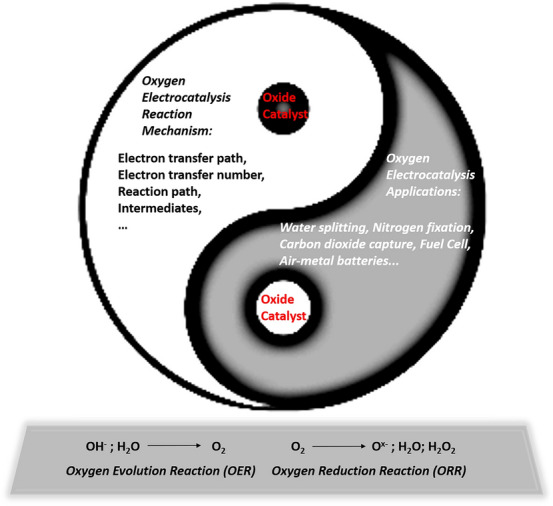

## Problem Statement

The growing energy demand caused by population increase has resulted in the accelerated use of fossil fuels and serious environmental consequences [1–5]. The depletion of fossil fuel reserves, combined with the urgent need to reduce greenhouse gas emissions in order to combat climate change, has inexorably pushed humans to transform the existing electricity generating grid into more renewable and sustainable alternative structures [6–10]. The difficulty in using renewable energy (such as solar, wind, and hydropower) is that it is intermittent, necessitating the development of energy storage and conversion technologies to deliver energy when it is needed [11–13]. Because of its high mass-energy density and absence of greenhouse gas emissions, hydrogen is a potential alternative energy carrier for replacing the existing fossil fuel grid [14–16]. The bulk of hydrogen generation is hampered by the costly and energy-intensive steam reforming of hydrocarbons, which are mostly derived from fossil fuels and emit large amounts of pollutants [17–20]. Cost-effective hydrogen production technologies are required for the adoption of renewable energy projects [17, 21–23]. Despite the technological challenges, electrochemical water splitting is a promising method of producing hydrogen-28]. Electricity may be stored in the chemical bond of H_2_ through electrochemical splitting of water, and electricity can be generated later by recombining H_2_ with O_2_ in a fuel cell [24–27]. In this respect, it is worth noting that the most prevalent element in the Earth's crust is oxygen [28–34]. Oxygen reduction is also the most essential reaction in life processes (e.g., biological respiration) and energy conversion devices (e.g., fuel cells). Water electrolysis and fuel cell technology with a high efficiency are needed to realize this plan. Because the oxygen evolution reaction (OER) at the anode is kinetically much slower than the hydrogen evolution reaction (HER) at the cathode, it dominates the efficiency of water electrolysis [35–37]. For fuel cells, the oxygen reduction reaction (ORR) is the slowest half-reaction mainly relying on rare noble metal catalysts [38–44]. Thus, it is important to conduct investigations on oxygen electrodes (OER and ORR).

Oxygen (O_2_) electrochemistry is the study of the electrocatalysis or reduction of molecular oxygen [45]. The oxygen evolution reaction (OER) is the generation of O_2_ molecules by removing electrons from water reactant, whereas O_2_ molecules combine with electrons to produce through oxygen reduction reaction (ORR). Because of its complexity and relevance in many practical technologies and industrial processes, oxygen electrocatalysis has been extensively explored. It is particularly important in numerous renewable energy technologies, including water electrolysis, fuel cells, and other industrial uses. Before catalyst design, it is necessary to understand the reaction path and reaction mechanism. Briefly speaking, OER is the electrochemical reaction that produces oxygen molecules via a series of coupled proton/electron steps. The reaction pathways are quite different in acidic and alkaline electrolytes [46, 47]. Hydroxyl groups (OH^−^) are oxidized in alkaline electrolytes and transformed into water (H_2_O) and oxygen molecules (O_2_) [48], while two water molecules (H_2_O) are oxidized in acidic electrolytes to produce four protons (H^+^) and one oxygen molecule (O_2_) [11, 49]. Due to the abundance of hydroxyl groups in the electrolyte, alkaline OER has more favorable reaction kinetics than acidic OER [50, 51]. For acidic OER, a high energy is needed to break the strong covalent O–H bond in H_2_O, which results in slow kinetics and worse OER performance [25, 52–54]. In spite of more favorable kinetics for alkaline OER, acidic OER is more applicable to commercialization because of the successful development and large-scale applications of proton exchange membrane (PEM) [55]. Thus, the promising electrocatalysts for OER should be not only active for oxygen thermodynamic reaction but also conquer the kinetic barriers. Meanwhile, ORR, another very important oxygen electrocatalysis, has been widely investigated. ORR in aqueous solutions is mostly accomplished through two pathways: the direct 4-electron reduction or 2-electron reduction from O_2_ to water (H_2_O) or hydrogen peroxide (H_2_O_2_), respectively [56, 57]. There are multiple different reaction routes depending on the pH of the electrolyte and catalyst. The dissociative and associative routes are two putative response pathways for the ORR mechanism. The O–O bond is disrupted as O_2_ is adsorbed onto two metal active sites through the dissociative pathway. As a result, no peroxide intermediate is produced. The O–O bond in oxygen molecules is difficult to break, making the dissociative pathway unlikely for most catalysts. For the associative route, the O_2_ is adsorbed onto a single metal site, which makes it easier to break the O–O bond and give rise to the formation of a peroxide intermediate. Evaluating the OER and ORR reaction processes can lead to the design of the optimal catalyst by identifying the catalyst characteristics for maximal electrocatalysis activity and selecting the target chemicals [45, 58–60].

An excellent oxygen electrocatalyst should provide adequate binding to oxygen species (neither too strong nor too weak). Until now, noble metal-based materials such as Ir, Ru, and Pt have shown to be the ideal catalysts for the oxygen electrocatalysis [61–64]. The high cost and scarcity of these precious metals limit their widespread use. From the standpoint of commercialization, it is not just the high cost of noble metal components that causes economic strain, but also the increased costs incurred due to the difficulties of manufacturing multiple cathode–anode products and potential cross-contaminations [65, 66]. As a result, a lot of effort has gone into developing efficient and low-cost oxygen electrocatalysts for OER/ORR [67, 68]. Transition metal oxides not belonging to the platinum group metals are a low-cost material type providing a d orbital for the binding of oxygen species. Among the transition metal oxide catalysts, the perovskite-like catalysts, such as single perovskites (ABO_3_) [69–71], double perovskites (A_2_B_2_O_6_) [72, 73], Ruddlesden–Popper perovskites (A_2_BO_4_) [74–76], and pyrochlore-type oxides (A_2_B_2_O_7_) [77], have gained recognition as materials for electrochemical catalysts due to the low cost of transition metals in the crystal [78], robust skeletal structure, intrinsic nature of harboring oxygen vacancies, electronic structural versatility, and compositional flexibility [25]. Perovskite-like oxides can work as oxygen conductors, proton conductors, or mixed ionic-electronic conductors depending on the occupancy of various metal ions in the A and B sites; thus, it has been employed as functional materials in energy and environmental domains during the last few decades [79–85]. To create effective perovskite-like electrocatalysts, researchers should focus more on the fundamental principles of developing perovskite materials and the relationship between catalysts and reaction processes [86–90]. Because electrocatalytic reactions generally entail the transfer of electrons, oxide catalysts with high electronic and ionic conductivity likely have superior electrocatalytic performance [90, 91]. The high electrical conductivity of catalysts reduces the ohmic losses and enhances the catalytic efficiencies in electrochemical devices. Based on the fundamental understanding of reaction paths, research has been performed on the development of various highly active, low-cost perovskite-like oxide catalysts for oxygen electrocatalysis and related applications [86, 92–94].

## General Principles of Oxygen Electrolysis

### Reaction Mechanism of Oxygen Evolution

**Fig. 1:**
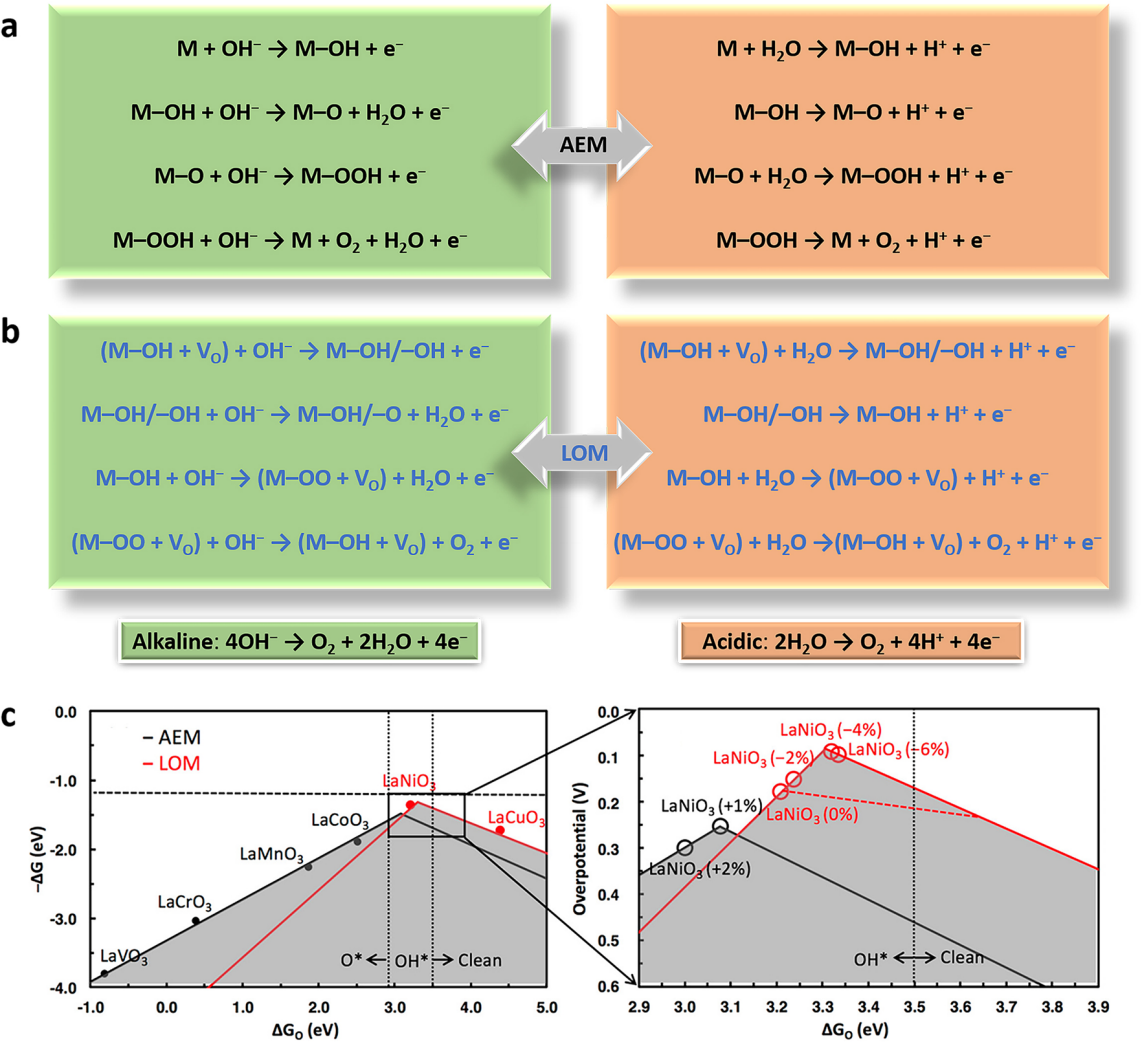
Reaction pathways for the OER in acidic and alkaline media via **a** AEM, and **b** LOM. **c** The overall OER activity volcano considers both AEM (black) and LOM (red) [97]

The OER is a complex reaction that involves the transfer of four electrons [69, 95]. The conventional adsorbate evolution-dominated mechanism (AEM) proceeds via a sequence of concerted electron–proton transfers on the transition metal active centers, and binding of the adsorbed oxygen intermediates should be neither too strong nor too weak, leading to (△G_O*_-△G_HO*_) of 1.6 eV, according to the Sabatier’s principle [78, 96]. According to the AEM mechanism, the intermediate M–OH is initially formed by a one-electron oxidation of hydroxide anion on the basis of surface metal sites (M) as a catalytically active site. Then, the M–OH is converted to M–O via the electron transfer and proton coupling step. When a hydroxide anion undergoes one-electron oxidation, M–O converts to M–OOH, which then undergoes another electron transfer and proton coupling process to produce O_2_ molecules. In contrast to the OER process in alkaline media, the initial step in the acidic media is the adsorption of H_2_O on M (Fig. 1a), followed by the water dissociation to form M-OH and second proton release to form M–O. Following that, another water molecule nucleophilically attacks M–O, culminating in the generation of M–OOH.

In recent years, a novel mechanism based on the redox chemistry of lattice oxygen anions, known as the lattice oxygen-mediated mechanism (LOM), which differs from the well-known AEM, has been proposed. This mechanism is validated by ^18^O isotope detection of the reaction product as well as density functional theory calculations and entails the direct participation of oxygen anions from the oxide catalysts lattice as an active intermediate in the OER [89, 97–100]. LOM can bypass the scaling limitations of the conventional AEM and lead to better OER kinetics. For example, LOM in alkaline electrolytes (Fig. 1b), the hydroxyl is adsorbed on the oxygen vacancy-coordinated metal site. Then, another hydroxyl adsorbs at the V_O_ site, following a dehydrogenation step. The hydroxyl is difficult to dehydrogenate directly, resulting in the formation of a transition state. Finally, the original state M–OH/–V_O_ is regained after releasing the oxygen and filling the hydroxyl. The acidic LOM process is like the alkaline one, with the main difference being the absence of ${\text{OH}}^{-}$ and the creation of ${\text{H}}^{+}$ in each stage. The oxygen from the catalyst engages directly in OER in the LOM process, resulting in more effective catalysis.

A fundamental contrast between LOM and AEM is the origin of the oxygen molecules, which are created not only from water molecules but also from lattice oxygen of the oxides [78]. LOM is thought to be more effective than AEM [97, 101], because AEM is restricted by the theoretical potential (0.37 V), but LOM with direct O–O coupling can avoid scaling relation limits [78]. It should be noted that entirely denying AEM is not realistic. AEM is constrained by the oxygenated intermediates, which has prompted the investigation on LOM. LOM, on the other hand, is often associated with significant V_o_ formation and metal dissolution, resulting in unstable crystals, whereas AEM catalysts, in theory, do not experience this extreme surface reconstruction [102]. AEM and LOM frequently occur concurrently, creating conflict between these two systems. As a result, balancing stability and activity in OER remain an important challenge in the quest to discover efficient and stable catalysts. Recently, in ABO_3_ electrocatalysts, adjusting the B-site metals, enhancing the covalency of metal–oxygen, and generating bulk oxygen vacancies were shown to improve the process of transition from the AEM mechanism to the LOM mechanism [89]. Moreover, Kolpak’s group explored a range of perovskite catalysts, such as LaVO_3_, LaCrO_3_, LaMnO_3_, LaCoO_3_, LaNiO_3_, and LaCuO_3_, to study the theoretical overpotential (Fig. 1c) [97]. AEM is ubiquitous in the OER activity volcano trend on ABO_3_ perovskites, but LOM is reliant on A cation sites.

### Reaction Mechanism of Oxygen Reduction

**Fig. 2:**
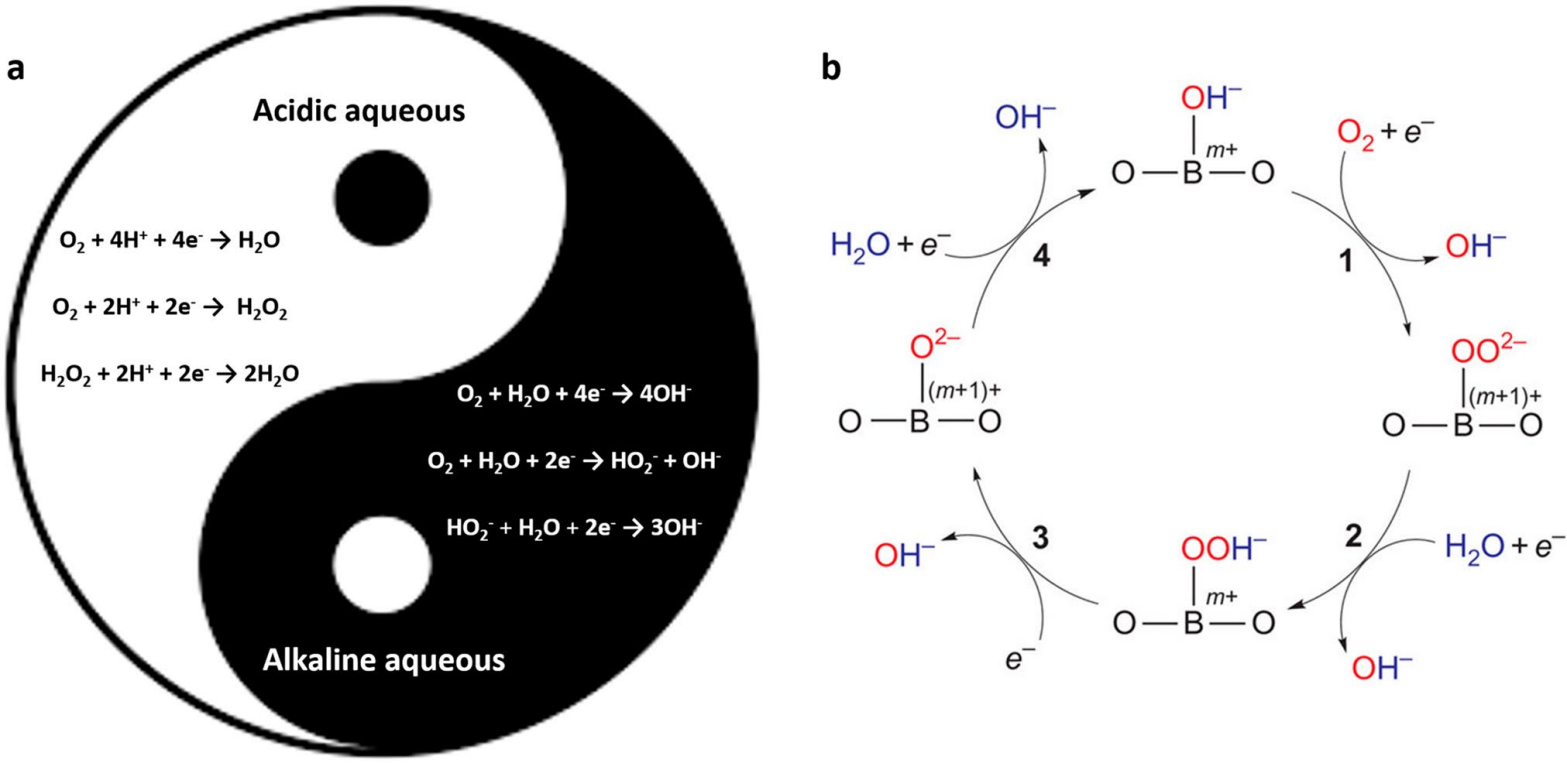
**a** Normal reaction pathways for the ORR in acidic and alkaline media. **b** Proposed ORR mechanism on oxide catalysts. The ORR process via four steps: 1, surface hydroxide displacement; 2, surface peroxide formation; 3, surface oxide formation; 4, surface hydroxide regeneration [104]

Although the ORR has been extensively studied in conjunction with the creation of fuel cells, the precise reaction mechanism is still being debated [103]. Figure 2a depicts the overall four-electron reaction. The first electron transfer steps to adsorbed oxygen species, with or without fast proton transfer, are commonly recognized as the RDS on noble metal (e.g., commercial Pt-containing) catalyst surfaces. The initial electron transfer step to O_2_ (${\text{O}}_{2}+ {e}^{-} \to {\text{O}}_{2}^{-}$) is surface sensitive, which displaces H_2_O from the electrode surface and inhibits the formation of HO_2_^−^ species. O_2_ is a stable molecule before decomposing into O_2_^−^ and HO_2_^−^; thus, it may be observed in the cyclic voltammograms via reverse sweep. The pH changes resulted in a drop in the overpotential from 1.53 to 0.7 V with pH values changing from 0 to 14 for O_2_/O_2_^−^, whereas the standard reduction potential decreased from 1.229 to 0.401 V for H_2_O/OH^−^. Conversely, the ORR reaction pathways on transition metal oxide surfaces follow a different logic than those on precious metal surfaces. To achieve total oxygen coordination, surface cations of transition metal oxides interact with the oxygen of H_2_O. The hydrogen atoms of H_2_O are spread throughout the catalyst surface, as the protonation is charge compensated by the reduction of cation at surface such as Fe^3+^, Co^3+^, Mn^4+^, to produce OH^−^ (Fig. 2b). Inorganic chemistry concepts help explain the interaction of oxide catalysts with O_2_ such as crystal field theory and molecular orbitals [104].

## Overview of Oxide Catalyst for Oxygen Electrocatalysis

**Fig. 3:**
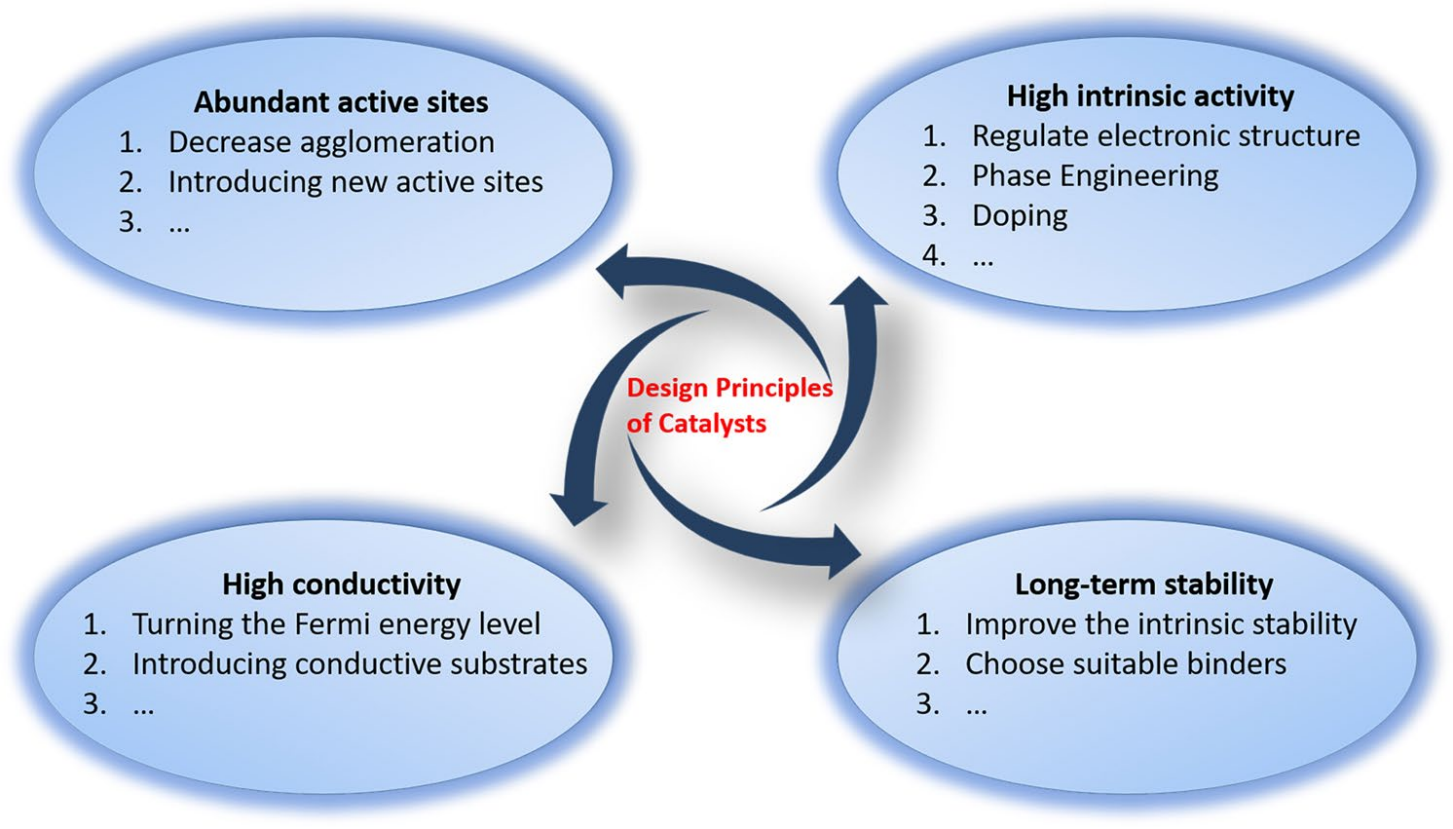
Design principles of oxide electrocatalysts for OER/ORR

The development of active, low-cost oxygen electrocatalysts is crucial to addressing the efficiency issue. The catalyst design must be predicated on a solid understanding of the OER and ORR mechanism and the source of the reaction over potential. The development of high-performance electrocatalysts follows the four general principles: abundant electroactive sites; high intrinsic catalytic activity; high electrical conductivity; long-term performance stability (Fig. 3). Based on these principles, it is possible to screen catalyst candidates by regulating the electronic structure [83], morphology [105, 106], crystallinity [107], foreign elements doping into the lattice [35, 108], vacancies [109], strains [110, 111], e_g_ orbital occupancy [69, 104, 112], metal–oxygen covalency [45, 113], controlling and engineering the interface [114, 115], etc. Moreover, controlling the reaction path of OER/ORR would directly adjust the OER/ORR performance, for example adjusting the redox chemistry of lattice oxygen anions to balance the proportion of LOM and AEM would facilitate the OER process [89, 116].

### Substitution of Foreign Elements

It is well known that introducing heteroatoms is a simple and efficient way to accelerate sluggish reaction kinetics [117, 118]. Table 1 summarizes the substitution of foreign elements for the oxygen electrocatalysis. Doping changes the binding energy of intermediates such as *O–OH, *OH, and *O by modifying the electronic structure. As a result, as compared to alternative ways for enhancing OER/ORR activities, doping provides an efficient means of achieving long-term stability with large-scale activity. By integrating extra elements, doping produces ionizable species inside the host material. The ionizable species may cause a change in the Fermi level (*E*_F_), allowing for better catalytic activity in carrier transport [119]. Nowadays, the current commercial ORR catalyst is still Pt/C, while commercial acidic OER catalysts are mainly RuO_2_ and IrO_2_. Thus, catalysts are usually compared to these commercial catalysts as standard from activity and durability aspects. In this section, we will go through the effects of doping strategy in the oxide catalysts for oxygen electrocatalysis.

**Table 1:** Representative summary of substitution of foreign elements for the oxygen electrocatalysis

Catalyst	Foreign element	Applications	Electrolyte	Performance	Refs
NiCoFeO	Fe	OER	1 M NaOH	1.43 V vs. RHE at 10 mA cm^−2^	[117]
Cr_0.6_Ru_0.4_O_2_	Cr	OER	0.5 M H_2_SO_4_	1.41 V vs. RHE at 10 mA cm^−2^	[120]
Cu-RuO_2_	Cu	OER	0.5 M H_2_SO_4_	1.42 V vs. RHE at 10 mA cm^−2^	[121]
Mn-RuO_2_	Mn	OER	0.5 M H_2_SO_4_	1.39 V vs. RHE at 10 mA cm^−2^	[122]
Ce_x_-IrO_2_	Ce	OER	0.5 M H_2_SO_4_	1.45 V vs. RHE at 10 mA cm^−2^	[123]
Mg-RuO_2_	Mg	OER	0.5 M H_2_SO_4_	1.46 V vs. RHE at 10 mA cm^−2^	[124]
La_0.6_Sr_0.4_CoO_3_	Sr	OER	0.1 M KOH	1.53 V vs. RHE at 0.19 mA mg_oxide_^−1^	[125]
La_0.3_–5582	La	OER and ORR	0.1 M KOH		[126]
PrBa_1−x_Sr_x_Co_2_O_5+δ_	Sr	OER and ORR	0.1 M KOH	1.52 V vs. RHE at 10 mA cm^−2^ for OER; 0.75 V vs. RHE, onset potential for ORR	[127]
YCR	Co	OER	0.5 M H_2_SO_4_	1.50 V vs. RHE at 10 mA cm^−2^	[128]
Sr_0.95_Ce_0.05_Fe_0.9_Ni_0.1_O_3_	Ce/Ni	OER	0.1 M KOH	1.57 V vs. RHE at 10 mA cm^−2^	[129]
Co-RuO_2_	Co	OER and ORR	0.1 M KOH	1.49 V vs. RHE at 10 mA cm^−2^ for OER; 0.82 V vs. RHE, half-wave potential for ORR	[130]

**Fig. 4:**
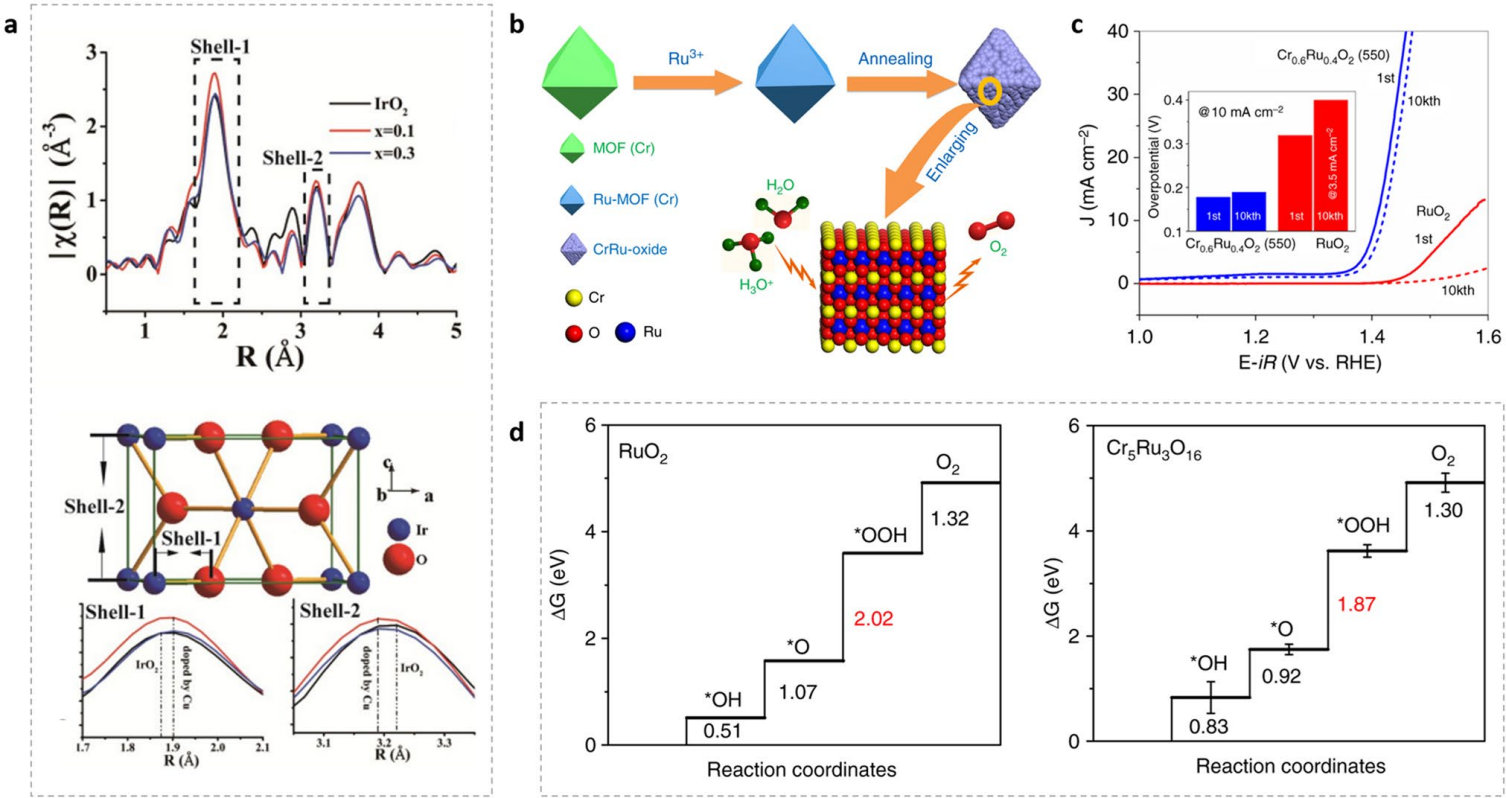
**a** Fourier transforms of the k 3 -normalized Ir-LIII edge EXAFS for Cu_x_Ir_1−x_O_δ_ compositions (up), and the sketch of one unit cell of IrO_2_ (up). The shell-1 formed due to an Ir-O bond, and shell-2 formed due to an Ir-Ir bond [133]. **b** The synthesis of Cr_0.6_Ru_0.4_O_2_ electrocatalysts for OER use in acid conditions is depicted schematically. **(c)** LSVs of Cr_0.6_Ru_0.4_O_2_ (550) and commercial RuO_2_ for the first and 10,000th cycle. **d** The calculated free energy diagrams for RuO_2_ and Cr_5_Ru_3_O_16_ [120]

Researchers doped RuO_2_ with a trace of Ir (Ru_x_Ir_1−x_O_2_) and discovered a considerable gain in stability while compromising OER performance [131]. Earth abundant metals substitution such as Co [132], Cu [121, 133], Mn [122], Ni [132], Ce [123], Mg [124], W [134], Cr [120], and Zn [135] is another method of increasing OER activity with reduced overall noble metal concentration. For instance, after Cu doping, Cu_0.3_Ir_0.7_O_δ_ exhibited enhanced OER activity in acidic, neutral, and alkaline conditions compared to pure IrO_2_ [133]. It was determined that increasing the Jahn–Teller effect in the IrO_6_ and CuO_6_ octahedral (Fig. 4a) structure would enhance the lift degeneracy of the e_g_ and t_2g_ orbitals and thus improve the catalysis performance. It minimizes the gap in free energy between ΔG_2_ and ΔG_3_ from density functional calculations (DFT), resulting in a reduced theoretical overpotential compared to IrO_2_ [133]. Recently, Chen's group reported metal–organic framework (MOF)-derived Cr_0.6_Ru_0.4_O_2_ for acidic OER [120]. RuCl_3_ was first loaded into the pores of MIL-101 (Cr) by impregnation. The resulting RuCl_3_-MIL-101 (Cr) was annealed in air for 4 h between 450 and 600 °C to produce Cr_0.6_Ru_0.4_O_2_ catalyst (Fig. 4b). Cr_0.6_Ru_0.4_O_2_ (550 °C) displays not only excellent OER activity, but also strong stability with only 11 mV overpotential decrease after 10,000 cycles at 10 mA cm^−2^ (Fig. 4c). DFT simulations showed an additional mechanism for the high OER performance. The RDS at the Ru site on the Cr_5_Ru_3_O_16_ surface was determined to be 1.87 eV, 0.15 eV lower than RDS on the RuO_2_ (2.02 eV) (Fig. 4d) [120].

**Fig. 5:**
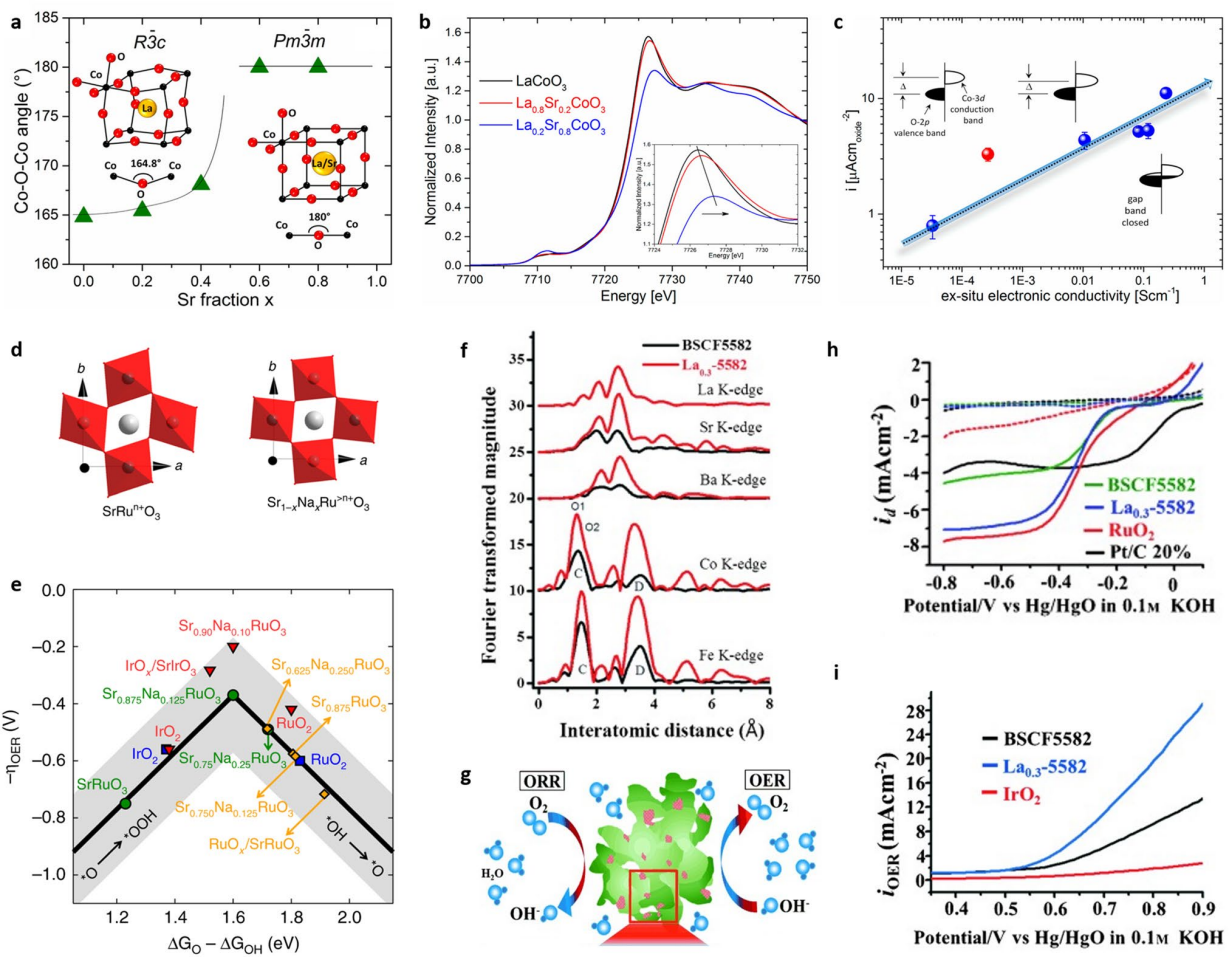
**a** Evolution of Co–O–Co angle as a function of the Sr fraction [125]. **b** Co K-edge XANES spectra of the La_1–x_Sr_x_CoO_3_ at room temperature [125]. **c** Current density (μA cm_oxide_^–2^) as a function of the ex situ electronic conductivity of the La_1–x_Sr_x_CoO_3_ series; the red circle represents the SrCoO_2.5_ [125]. **d** Octahedral distortion in SrRuO_3_ compared to Na-doped Sr_1-x_Na_x_RuO_3_ [138]. **e** OER Volcano-type activity plot [138]. **f** The radial distribution function for the all atomic EXAFS spectra for BSCF5582 and La_0.3_–5582. **g** The rhombohedral phase LaCoO_3_ grains are separated on the surface of cubic-based grains of parent La_0.3_–5582. LSV of **h** ORR and **i** OER on BSCF5582 and La_0.3_–5582 compared to commercial catalysts [126]

It is also a universal strategy via doping for perovskite-like oxides to improve the OER/ORR activity and stability [75, 80]. The standard perovskite with the general formula ABO_3_, where A is either an alkaline earth metal or a rare earth metal, and B is a transition metal, is widely investigated according to doping strategy for oxygen electrocatalysis. Doping at the A-site, B-site, or even O-site to induce oxygen vacancies, which adjust the electronic structure of other parts in the perovskite, can be used to modify the OER/ORR activity. The oxidation state of transition metal affects metal–oxygen hybridization; therefore, the electronic behaviors of these perovskites depend on this parameter. Strongly electronegative transition metals exhibit high metal–oxygen hybridization, and the d-band center of the transition metal oxide gets closer to O 2*p* states as the electronegativity increases. However, in addition to metal–oxygen hybridization, cation doping also brings other effects on octahedral tilting and Jahn–Teller distortion, which all play important roles in determining the electrical characteristics of perovskite oxides [136, 137]. The A-site is not commonly regarded as the active site directly involved in the oxygen electrode processes. However, A-site cations may have an indirect effect on the perovskite's ORR/OER performance. Sr replacements in La_1−x_Sr_x_CoO_3_ are found to straighten the octahedral cage, align atoms along the Co–O–Co axis (Fig. 5a), and increase the average oxidation state of the Co cations (Fig. 5b). As a result, both electrical conductivity and activity toward the OER are significantly enhanced (Fig. 5c). According to DFT, the alignment of the Co–O–Co bonds and the oxidation of the Co cations enhance the overlap between the occupied O 2*p* valence bands and the unoccupied Co 3*d* conduction bands, explaining why conductivity improves as the amount of Sr increases [125]. Doping the Sr site with Na (Sr_1−x_Na_x_RuO_3_) improves the activity and durability of SrRuO_3_ perovskite, resulting in a decrease in octahedral distortion and an increase in the oxidation state (Fig. 5d) [138]. Although SrRuO_3_ binds reaction intermediates too strongly, Na doping of 12.5 at% leads in nearly optimal OER activity. The addition of Na increases the oxidation state of Ru, displacing positively charged O p-band and Ru d-band centers and weakening Ru-adsorbate bonds (Fig. 5e). The increased stability of Na-contained perovskites is due to the stability of Ru centers, which results in somewhat higher oxidation states, lower surface energy, greater dissolving potentials, and less deformed RuO_6_ octahedra [138]. In addition to the cations doping with lower valence, the cations with higher valence are also active for oxygen electrocatalysis. Recently, it has been reported that doping La into the A-site of BSCF5582 selectively causes local stress on the Co sublattice octahedron, which lead to a better ORR and OER performance [126]. From the EXAFS data (Fig. 5f), the peak intensities of La_0.3_–5582 are shown to be significantly higher than those of BSCF5582 in all atomic edges, indicating that La-doping promotes the stretch-up of atomic arrangement in the lattice. Meanwhile, the peak of the Co K-edge is divided into two peaks with different r spaces. It is reasonable to claim that the selective impact of the La-doping can cause local stress on the unit cell of the Co sub-lattice in a cubic perovskite structure, while the unit cell of the Fe-site sub-lattice is less affected, causing the simultaneous appearance of rhombohedral LaCoO_3_ aggregates and the comminution of particles (La doped BSCF5582) into smaller ones (Fig. 5g) [126]. In other words, the dynamic microstructure phenomena are caused by the implantation of A-site cations with La^3+^ (charge imbalance) and local stress on the Co-site sub-lattice with the cubic perovskite structure. After La-doping, the synthesized La_0.3_(Ba_0.5_Sr_0.5_)_0.7_Co_0.8_Fe_0.2_O_3-δ_ displayed excellent OER and ORR performance (Fig. 5h, i) [126]. Another effective higher valence ion is Co^4+^, which has been shown to possess good electrophilicity [139]. The OER activity of the perovskite is enhanced by the electrophilic surface Co^4+^, which makes it easier to generate surface O-OH and deprotonate surface-adsorbed OOH* species via an inductive, electron-withdrawing effect [139]. Adjusting the electronic surface structure of catalysts via doping can improve the non-electrochemical steps (adsorption and desorption processes) [104], of PrBa_1−x_Sr_x_Co_2_O_5+δ_ in which the surface-accumulated Co^4+^ was found to promote both OER and ORR as an excellent bi-functional catalyst [127]. The doping strategy is an effective method to adjust the surface structure of catalysts and then affect the interactions because of the modulated electron unsaturated state, for example, high oxidation status of metals sites displaying higher electrophilicity and stronger absorption efficiency/weaker desorption efficiency for OH^−^.

### e_g_ Orbital Occupancy

**Fig. 6:**
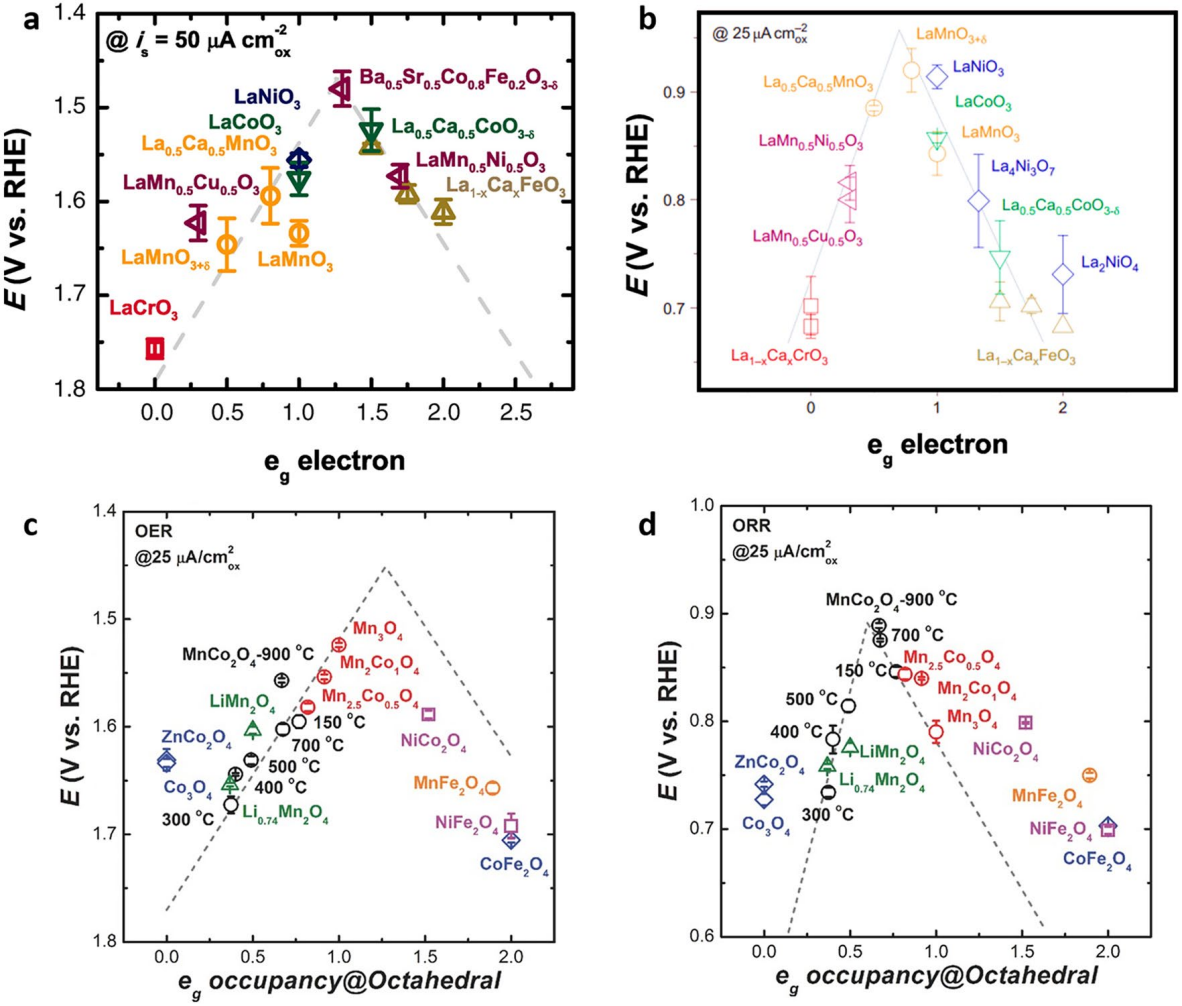
The relation between the **a** OER and **b** ORR catalytic activities and the occupancy of the e_g_-symmetry electron of the perovskite oxides [104, 142]. OER activity defined by the overpotentials at 50 μA cm_oxide_^−2^ of OER current; ORR activity defined by the overpotentials at 25 μA cm_oxide_^−2^ of ORR current. **c** OER and **d** ORR activity on various spinel oxides as a function of e_g_ occupancy of the active element at octahedral site [74]

Because the B-site directly participates in oxygen redox reactions, B-site substitution is a straightforward and effective way for regulating the ORR/OER activity of a perovskite catalyst [140, 141]. The discovery of a catalyst design approach that links material properties to catalytic activity by Shao-Horn's group speeds up the search for highly active and cheap transition-metal-oxide catalysts to replace precious metal catalysts (Ru, Ir, Pt) [104, 142]. The intrinsic OER activity of surface transition metal cations in an oxide exhibits a volcano-shaped dependence on 3d electron occupancy with an e_g_ symmetry. The best OER activity was predicted to occur at close to unity e_g_ occupancy, with strong covalency of transition metal–oxygen links (Fig. 6a) [142]. Shao-Horn's group demonstrates that the ORR activity of oxide catalysts is mostly related to the B-site metal–oxygen covalency, which serves as a secondary activity descriptor (Fig. 6b) [104]. Xu’s group did further descriptor work on OER and ORR of spinel oxides, including Mn_x_Co_3−x_O_4_, XCo/Fe_2_O_4_, and Li_x_Mn_2_O [74]. The e_g_ occupancy of the active cation at the octahedral site is the activity descriptor for spinels' ORR/OER, confirming the relevance of electron orbital filling in metal oxide catalysis (Fig. 6c, d) [74]. When two distinct cations occupy the same octahedral site, the e_g_ values is utilized. There is a four-step proton/electron-coupled reaction mechanism [143, 144], in which the binding strength of OER/ORR reaction intermediates is governed by e_g_ filling [104, 142]. The electrocatalysis activity of spinel oxides is summarized and compared to the e_g_ occupancy as-extracted. For instance, octahedral element filling and active element occupancy at the octahedral site simultaneously determine oxygen electrocatalysis performance, demonstrating the usefulness of this paradigm (e_g_ occupancy) to unify the ORR/OER activity prediction of spinel oxides (Fig. 6c, d) [74]. Changing the oxidation state [90, 145–147], or the spin state [148, 149] of a transition metal can control the e_g_ orbital occupation of its 3d electrons in the octahedral site.

**Fig. 7:**
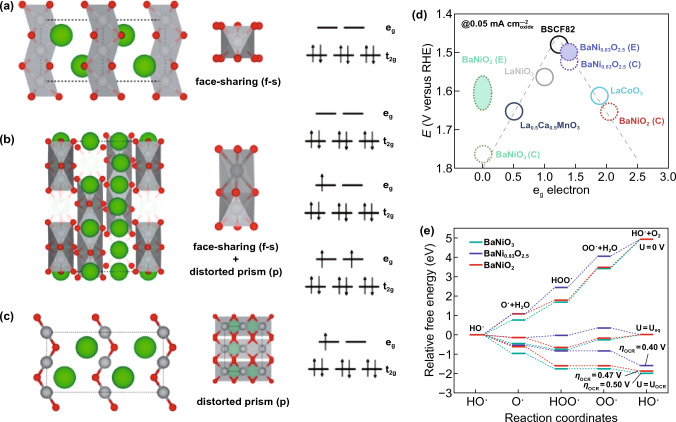
Schematics of phase transformation and the evidence of the OER activity of the BaNiO_3_. **a** Schematic of the BaNiO_3_ crystal structure with the interatomic distances calculated by DFT. **b** Schematic of the BaNi_0.83_O_2.5_ crystal structure with the interatomic distances calculated by DFT. **c** Schematic of the BaNiO_2_ crystal structure with the interatomic distances calculated by DFT. **d** The relation between the OER catalytic activity, defined by the overpotential at 0.05 mA cm^–2^_oxide_, of OER current, and the occupancy of the e_g_ electron of the transition metal. **e** Free energy diagrams of the BaNiO_3_, BaNi_0.83_O_2.5_, and BaNiO_2_ depending on the reaction coordinates [146]

Shao’s group developed SrM_0.9_Ti_0.1_O_3_ with different B-site transition metal elements for OER [150]. Both SrCo_0.9_Ti_0.1_O_3_ (SCT) and SrFe_0.9_Ti_0.1_O_3_ (SFT) exhibit enhanced OER activities compared to the original spinel, and also comparable to the Ba_0.5_Sr_0.5_Co_0.8_Fe_0.2_O_3_ (BSCF). SCT demonstrated greater operational stability than SFT, outperforming BSCF and IrO_2_ catalysts [150]. It was explained by the e_g_ filling which is 1.16 close to the standard 1 and the formation of redox-active oxygen deficiency [142]. Ciucci’s group introduced Nb into the Mn site of CaMnO_3_ (CMO) and treated the material with H_2_ to prepare CaMn_0.75_Nb_0.25_O_3−δ_ (H_2_-CMNO) which displayed dramatically enhanced OER/ORR activity compared to CMO [151]. This significant increase in OER/ORR activity can be attributed to Nb^5+^ doping inducing oxidation state changes on Mn from + 4 to + 3, which directly optimizes the e_g_ value close to unity to increase OH– adsorption [151]. Liu’s group also synthesized a double perovskite PrBa_0.5_Sr_0.5_Co_1.5_Fe_0.5_O_5+δ_ nanofiber as an efficient and robust catalyst for OER [152]. The co-doping strategy of Sr and Fe into PrBCo_2_O_5+δ_ is shown to be particularly successful in increasing the intrinsic activity about 5 times, which could be attributed to the favorable e_g_ electron filling. Shui’s group reported a hexagonal perovskite, BaNiO_3_ as a catalyst for OER in alkaline media, with an activity one order higher compared to IrO_2_ [146]. The underlying mechanism results from structural transition from BaNiO_3_ to BaNi_0.83_O_2.5_ (Ba_6_Ni_5_O_15_) throughout the OER cycle process (Fig. 7) [146]. The calculated e_g_-orbital fillings are 0 for BaNiO_3_ (Ni^4+^ (t_2g_)^6^(e_g_)^0^), 2 for BaNiO_2_ (Ni^2+^ (t_2g_)^6^(e_g_)^2^), and 1.4 for BaNi_0.83_O_2.5_ (Ni^2+^ (t_2g_)^6^(e_g_)^1^, Ni^3+^ (t_2g_)^6^(e_g_)^1^, and Ni^4+^ (t_2g_)^6^(e_g_)^2^), respectively (Fig. 7a–c) [146]. It agrees well with the principle put forward by Shao-Horn’s group [142], as BaNi_0.83_O_2.5_ displays the best OER activity and BaNiO_3_ the worst (Fig. 7d). The free energy diagrams of the BaNiO_3_, BaNi_0.83_O_2.5_, and BaNiO_2_ are shown in Fig. 7e. The underlying mechanism resulting in the highest OER activity of BaNi_0.83_O_2.5_ is as follows: the difference in free energies between OO* and HOO* on BaNi_0.83_O_2.5_ is maximized among the three perovskite oxides, as reflected by the smallest overpotential [146].

### Vacancies

**Fig. 8:**
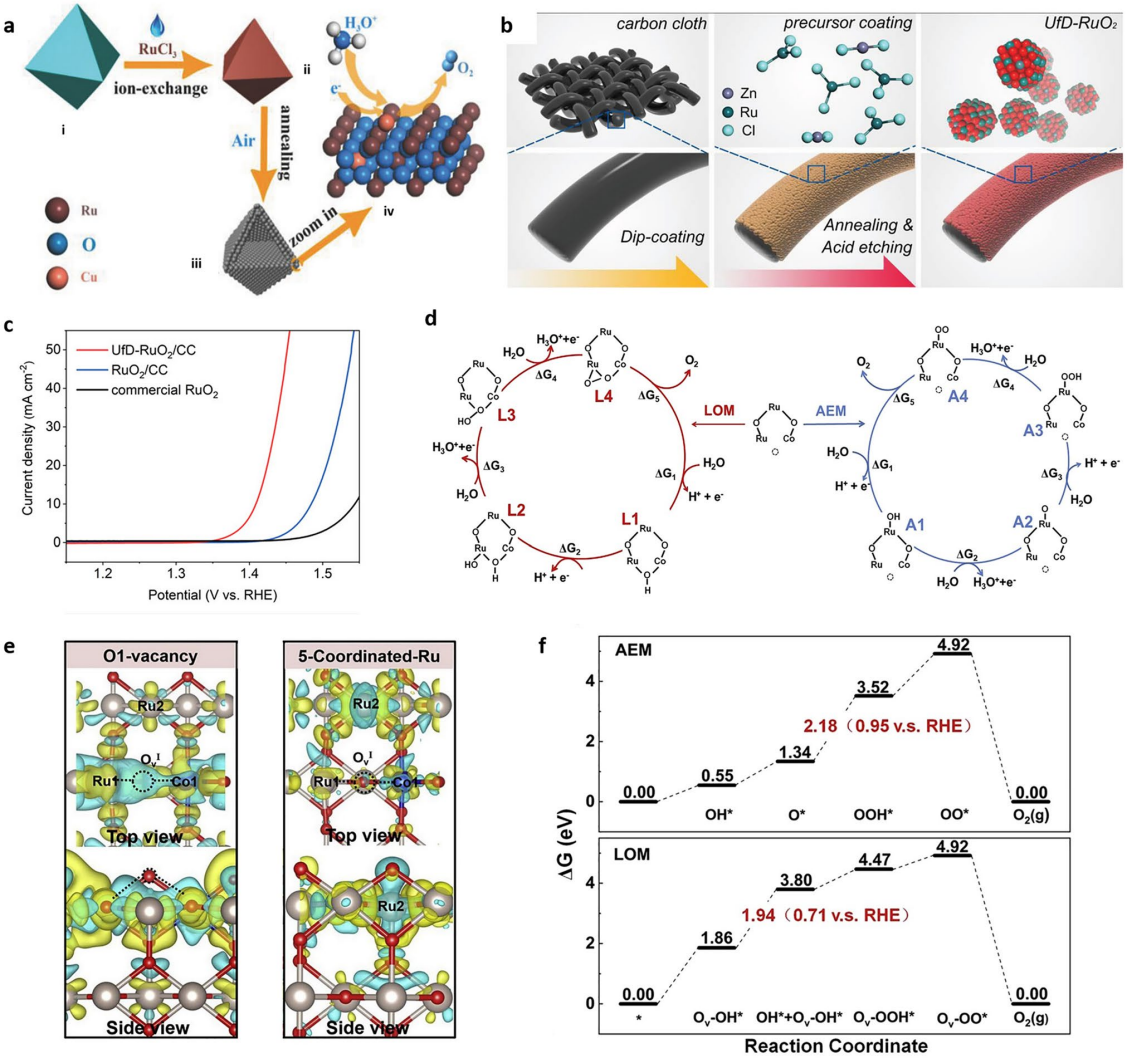
**a** The synthesis process and model of Cu-doped RuO_2_ hollow porous polyhedron are depicted schematically. (i) A Cu-BTC polyhedron, (ii) a Ru-exchanged MOF-derivative polyhedron, (iii) a hollow porous polyhedral aggregation of ultrasmall Cu-doped RuO_2_ nanoparticles, (iv) an expanded model of Cu-doped RuO_2_ as an electrocatalyst for OER in acidic environments [121]. **b** Illustration of the synthetic route for the UfD-RuO_2_/CC [135]. **c** OER polarization curves of samples synthesized (UfD-RuO_2_/CC, RuO_2_/CC, and commercial RuO_2_/CC) after capacitance-correction and iR-correction with the same mass loading in O_2_ saturated 0.5 m H_2_SO_4_ solution [135]. **d** Proposed LOM and AEM mechanisms. **e** The charge energy difference of A1 and L1 to illustrate the lower electron depletion on ${\text{O}}_{{\text{V}}}^{{\text{I}}}$ than Ru. **f** The free energy diagrams of the two mechanisms of LOM and AEM [163]

Defect engineering is a known strategy to design more active electrocatalysis for OER [153–155], hydrogen generation [55, 156], ORR [103, 157–159], nitrogen reduction reaction (NRR) [160, 161] and CO_2_ reduction (CRR) [162]. Another method for increasing OER activity and stability in acidic media is to produce defect-containing RuO_2_ by doping and dopant leaching. Cu-doped RuO_2_ porous nano-polyhedra generated from organic frameworks is likely one of the greatest examples of increasing OER activity via the production of oxygen vacancies (Fig. 8a) [121]. The bulk of the oxygen vacancies generated near Cu causes the adjacent Ru atom to become more negative and, as a result, shift the center of the O 2*p* band towards the Fermi level for greater OER activity. Tian et al. synthesized Zn-doped RuO_2_ and leached it out in acid to prepare ultrafine defective RuO_2_ (named UfD-RuO_2_/CC) (Fig. 8b), and the resulting catalyst demonstrated outstanding OER performance in acidic environments with 179 mV overpotential and 20 h stability at a current density of 10 mA cm^−2^. The strong catalytic activity is attributed to the synergistic impact of active sites and electronic structural tailoring (Fig. 8c) [135]. A cobalt-doped oxygen-defect Ru-based catalyst with 169 mV overpotential and 50 h endurance at 10 mA cm^−2^ current density was recently created. The significant increase in OER performance is attributable mostly to the oxygen vacancies and the changed electronic structure of the Co-doped RuO_2_, which employs a vacancy-related LOM path rather than an AEM path. To explore the role of oxygen vacancies in OER, the effect of oxygen vacancies on neighboring Ru atoms was investigated. The optimal lower free energy method was explored by comparing two competing OER processes via ${\text{O}}_{{\text{V}}}^{{\text{I}}}$ in LOM and 5-coordinated Ru in AEM (Fig. 8d). In both processes, five elementary steps are considered, including four electrochemical electron transfer steps (ΔG_1_∼ΔG_4_) and also the non-electrochemical O_2_ desorption step (ΔG_5_). For the first adsorption of the incoming H_2_O molecule, the intermediate L1 (${\text{O}}_{{\text{V}}}^{{\text{I}}}$–OH) in LOM and intermediate A1 (Ru–OH) in AEM were generated on ${\text{O}}_{{\text{V}}}^{{\text{I}}}$ and 5-coordinated Ru sites, respectively. The fact that L1 had a lower adsorption energy than A1 indicates that H_2_O preferred to react with the ${\text{O}}_{{\text{V}}}^{{\text{I}}}$ vacancy rather than the Ru site [163]. Due to the lower electron depletion of ${\text{O}}_{{\text{V}}}^{{\text{I}}}$ vacancy after binding to H_2_O, the following OER steps will process in LOM mechanism (Fig. 8e). Moreover, the effect of O vacancy on OER process was also further investigated on the rate-determining step (RDS) in both LOM and AEM (Fig. 8f). Also, the presence of O vacancies prevents the over-oxidation of Ru to soluble RuO_4_, which is thought to be the primary cause of the acidic instability of RuO_2_ electrocatalysts regulated by AEM [163].

**Fig. 9:**
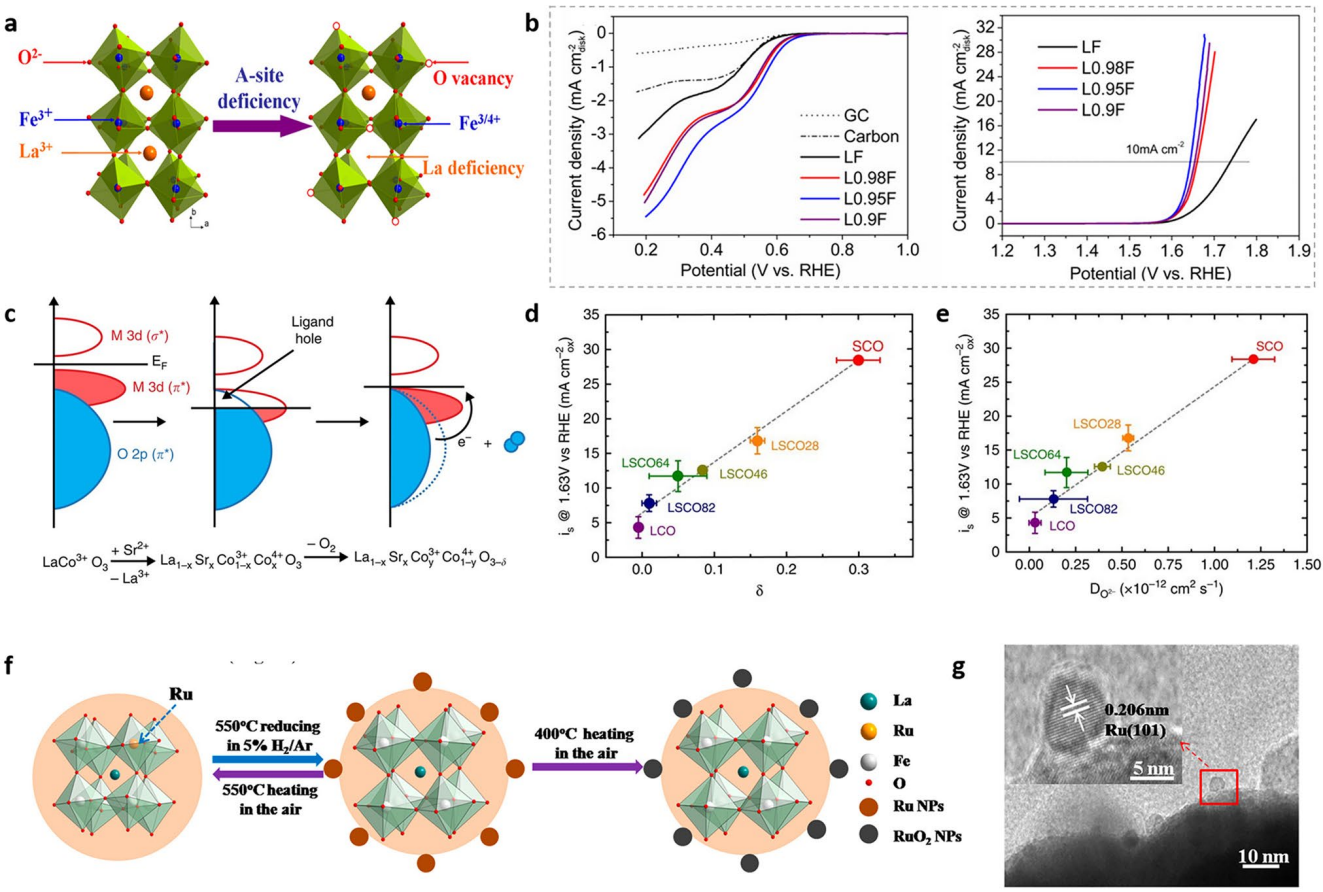
**a** Schematic illustration of the formation of oxygen vacancy and Fe^4+^ in A-site-deficient La_1–x_ FeO_3-δ_ perovskites [148]. **b** LSV curves for the ORR and OER on the RDE (1600 rpm) comprised of LF, L0.98F, L0.95F, and L0.9F catalysts in O^2−^saturated 0.1 M KOH solution [148]. **c** Relationship between oxygen vacancy concentration and Co–O bond covalency in La_1−x_Sr_x_CoO_3_ [167]. **d** Correlation of oxygen evolution activity with the vacancy parameter δ [167]. **e** Correlation of oxygen evolution activity with the oxygen ion diffusion rate [167]. **f** Lattice structures of LFRO, Ru/LFRO, and RuO_2_/LFRO. **g** TEM image of LFRO-550H, inset is the exsolution of a Ru nanoparticle [168]

Creating an A-site shortage in spinels is one method of creating oxygen vacancies to boost oxygen electrocatalysis [164]. Shao’s group presented a simple and effective method for creating A-site cation deficiency into LaFeO_3_ perovskite to increase ORR and OER electrocatalytic activity in alkaline solutions, but the enhancement is more significant for OER than for ORR (Fig. 9a, b) [148]. The increase is due to the formation of surface oxygen vacancies and a trace amount of Fe^4+^ species, as proven via XPS as the slight positive shift of Fe 2*p*_3/2_ peak could be observed for La_1−x_FeO_3−δ_ suggesting the presence of iron in a higher oxidation state (+ 4). Mössbauer spectroscopy is sensitive to iron and is commonly used to investigate iron's electrical structure. Furthermore, the pristine LF revealed a single Fe^3+^ sextet component with strong lines in the Mössbauer spectrum, whereas extra Fe^4+^ occurred in the A-site cation deficient La_1−x_FeO_3−δ_ [148]. The significance of oxygen vacancy defects, which allow crystalline oxygen to be mobile at the surface of perovskites, has hitherto gone unnoticed. How much the stoichiometry of oxygen in the crystal structure of perovskites deviates from the nominal value of 3 for the formula ABO_3_ influences both the lability of surface oxygen and the underlying electronic structure of these materials [165, 166]. The degree of vacancy generation depends on how close together the 2*p* band of oxygen and the 3*d* band of the metal are in the crystal, with more covalent systems displaying larger vacancy concentrations as shown in Fig. 9c [167]. The *d* orbitals of the Co ion have a higher overlap with the *s* and *p* orbitals of the O^2−^ ion as the oxidation state of Co increases, resulting in the creation of the π* and σ* bands. Stevenson's group has introduced a number of cobaltite perovskites in which the covalency of the Co–O bond and the amount of oxygen vacancies are controlled by the substitution of Sr^2+^ into La_1−x_Sr_x_CoO_3−δ_ [167]. When there is sufficient Co 3*d* and O 2*p* band overlap, vacancies are produced, which is indicative of the underlying electronic structure in the vacancy parameter (Fig. 9d). The vacancy would then affect oxygen diffusion rate, which is directly correlated with OER performance (Fig. 9e) [167]. Another strategy is to create cation deficiencies to adjust the electronic structure of other cations and the overlap of the d orbital of the metal and the 2*p* orbital from oxygen. Jiang et al. synthesized a RuO_2_/LFRO composite by the exsolution of a low Ru-substituted A-site deficient perovskite, La_0.9_Fe_0.92_Ru_0.08_O_3_ (LFRO) [168]. In this procedure, pure Ru NPs are exsolved from LFRO through a heat treatment in 5% H_2_/Ar at a relatively low temperature (Fig. 9f, g). Next, the exsolved Ru NPs were converted into RuO_2_ for the oxygen evolution reaction (OER). In comparison with the pure LFRO, the RuO_2_/LFRO composite had a strong OER performance, which was primarily due to the production of electrochemically active RuO_2_ NPs and the enhancement of the electrical conductivity [168]. Furthermore, the exsolution is a reversible process, and by heating at 550 °C in air, the exsolved Ru NPs “redissolve” into the perovskite lattice [168]. The in situ exsolution from perovskite oxide catalyst can also be used to synthesize electrocatalysts, for the hydrogen evolution reaction (HER). Zhu et al. reported that the La_0.4_Sr_0.4_Ti_0.9_O_3_ promoted the water dissociation and in situ generated Ni nanoparticles favor hydrogen adsorption for the recombination into H_2_ [169]. Table 2 summarizes some very important references with introducing vacancies for the oxygen electrocatalysis.

**Table 2:** Representative summary of introducing vacancies for the oxygen electrocatalysis

Catalyst	Vacancies	Applications	Electrolyte	Performance	Refs
Ru_1−x_O_2_	Ru	OER	0.5 M H_2_SO_4_	1.41 V vs. RHE at 10 mA cm^−2^	[135]
Co_0.11_Ru_0.89_O_2-δ_	O	OER	0.5 M H_2_SO_4_	1.41 V vs. RHE at 10 mA cm^−2^	[163]
La_0.8_Sr_0.2_CoO_3-δ_	O	OER	0.1 M KOH	8 mA cm^−2^_oxide_ at 1.63 V vs. RHE	[167]
SrCoO_3-δ_	O	OER	0.1 M KOH	28 mA cm^−2^_oxide_ at 1.63 V vs. RHE	[167]
La_0.9_Fe_0.92_Ru_0.08_O_3_	La	OER	0.1 M KOH	1.65 V vs. RHE at 10 mA cm^−2^	[168]
SrFe_0.9_Si_0.1_O_3_	O	OER	0.1 M KOH	1 mA cm^−2^_oxide_ at 1.63 V vs. RHE at	[170]
SrCo_0.9_Ru_0.1_O_3_	O	OER	0.1 M KOH	1.59 V vs. RHE at 10 mA cm^−2^	[35]
La_0.95_FeO_3_	La	OER	0.1 M KOH	1.64 V vs. RHE at 10 mA cm^−2^	[148]
La_0.95_FeO_3_	La	ORR	0.1 M KOH	1.43 mA cm^−2^_oxide_ at 0.25 V vs. RHE	[148]

### Strain

**Fig. 10:**
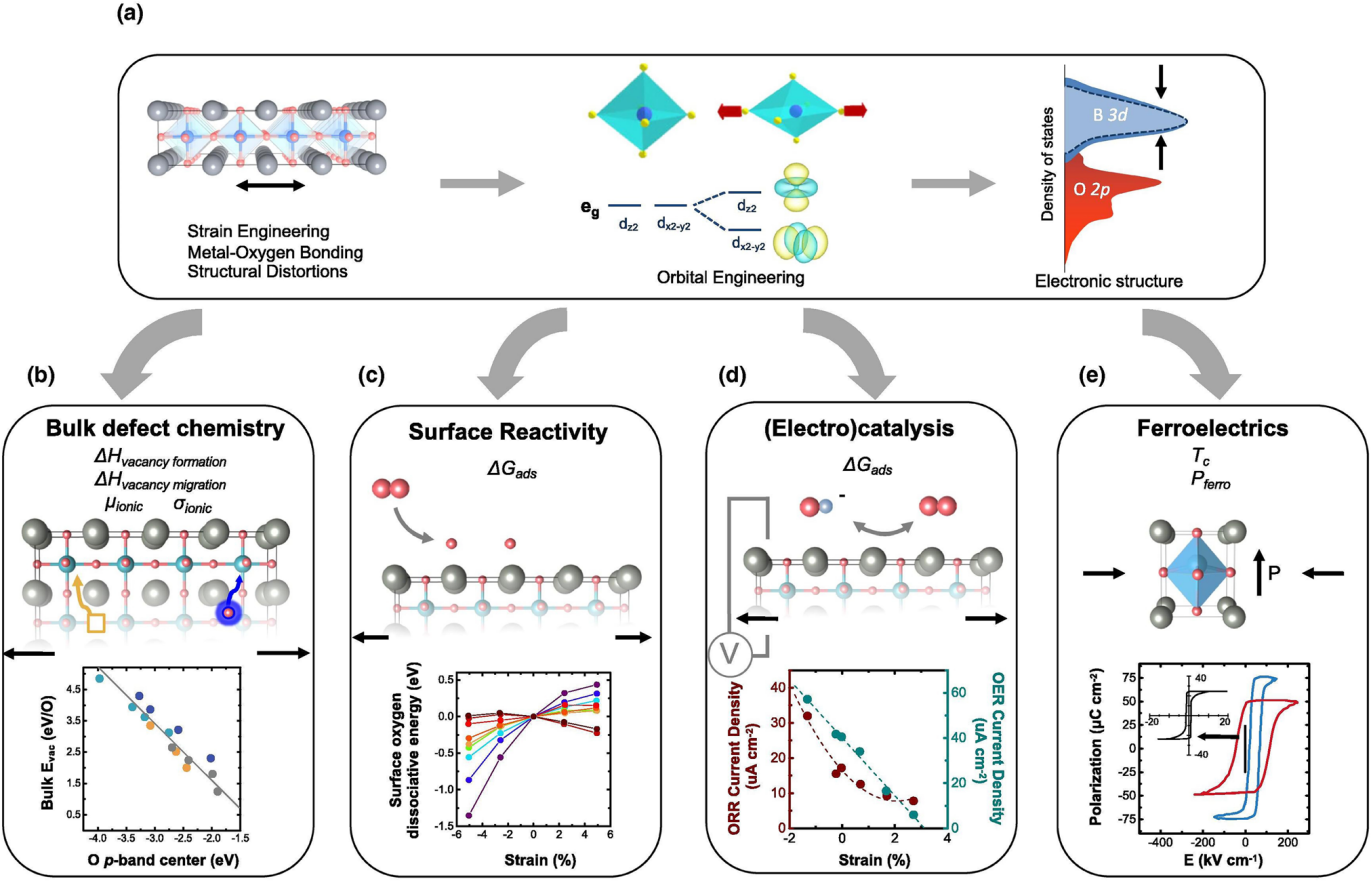
Interplay between strain in oxides, modification of electronic structure, and the resulting effect on oxide functionalities [172]. **a** Strain-induced structural modification divides previously degenerate energy levels due to symmetry-breaking, whilst changes in metal–oxygen orbital overlap impact the widening of d-states in the electronic density of states [172]. **b** Correlation between raising the oxygen 2p-band center and decreasing the bulk oxygen vacancy formation energy in perovskite oxides [177]. **c** Dependence of the oxygen dissociation energy for the BO_2_-terminated lanthanides [175]. **d** Relationship between the room temperature ORR at 0.823 V_RHE_ and OER at 1.623 V_RHE_ for LaNiO_3_ under various strain effects in 0.1 M KOH [110]. **e** Polarization behavior of single-crystal BaTiO_3_ versus BaTiO_3_ grown on DyScO_3_ and GdScO_3_ [179]

Strain engineering is an effective method for modifying the metal–oxygen binding energy [39, 171, 172]. The electrical structure can be tuned by lattice strain generated by lattice vacancies, distortion, or mismatch. Tensile strain and compressive strain are types of lattice strain that, under an octahedral coordination, for instance, promote the filling of in-plane (d_x2−y2_) and out-of-plane (d_z2_) orbitals, respectively [25]. Dr. Pesquera’s group disclosed the effects of symmetry breaking at free surfaces of ABO_3_ perovskite epitaxial films and show that it can be combined with substrate-induced epitaxial strain to tailor at will the electron occupancy of in-plane and out-of-plane surface electronic orbitals [173]. Modifying interatomic lengths has been widely studied in the context of strain engineering. The electronic structure of cubic ABO_3_ perovskite is significantly impacted by the octahedral structure, such as B-O bond lengths (Fig. 10a) [172, 174]. Strain has an impact on both the electronic properties and chemical properties. Systematic DFT calculations across the first-row transition metal perovskite oxides in the idealized cubic perovskite structure show that, for early transition metals, increasing the d-band width results in a higher d-band center relative to the Fermi level. These calculations assume constant d-band electron filling under strain and approximate the d-band as a rectangular form [175]. However, d-band widening causes a lower d-band center because of the higher d-band filling of late transition metals [175]. For instance, computations on relaxed La_0.6_Sr_0.4_CoO_3_ structures showed that in the region of -3% to 3% biaxial strain, tensile strain induced an upshift of the O 2*p*-band center in relation to the Fermi level [176]. The O 2*p*-band center thus establishes a relationship between effects of strain and a variety of physical and chemical characteristics. In addition to electrical factors like work function, the position of the O 2*p*-band center relative to the Fermi level is also related to bulk oxygen vacancy formation energy in lanthanide perovskites (Fig. 10b) [177]. Because of the strain's impacts on the d-band and O 2*p*-band centers and variations in metal–oxygen overlap, strain affects surface properties like oxygen dissociative adsorption energy. Therefore, strain regulation can affect the oxygenated species during the electrocatalysis, which improves the rate-determining step of oxygen electrocatalysis (e.g., HOO* → O* in acidic OER). Theory indicates that under highly strained conditions, tensile strain can diminish the dissociation of surface oxygen relative to bulk for early transition metals (Sc-Co), while having the reverse effect for later transition metals (Ni-Cu) (Fig. 10c) [175]. As a result, strain has an influence on electrocatalysis performance, with compressively stretched LaNiO_3_ catalysts displaying increased OER/ORR activity (Fig. 10d) [110]. Recently, it has been reported that spintronic and ferroelectric polarization regulation enhances the oxygen evolution efficiency of multiferroic oxides [178]. Thus, strain effects on ferroelectric polarization are also a promising method to adjust the oxygen electrocatalysis. As expected, the cumulative effects of strain result in a significant increase in ferroelectricity in strained BaTiO_3_ relative to the unstrained state (Fig. 10e) [179].

**Fig. 11:**
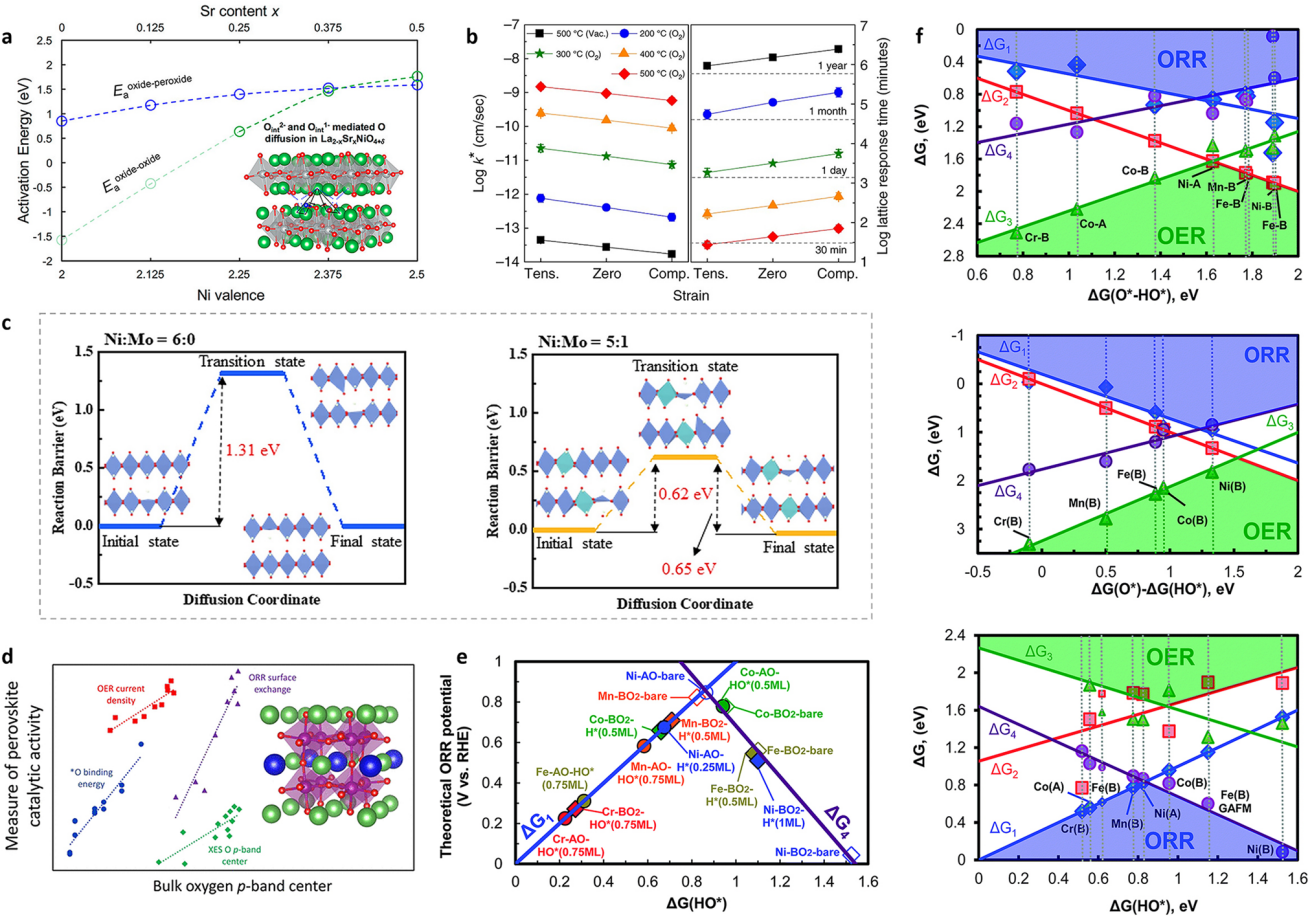
**a** Activation energies of the oxide–oxide and oxide–peroxide diffusion mechanisms [184]. **b** Strain-dependent oxygen kinetics of La_1.85_Sr_0.15_CuO_4_. Variation of predicted oxygen surface exchange rate k* and time required to incorporate oxygen under different annealing conditions and strain states [185]. **c** Diffusion pathway (insets) and the corresponding diffusion barrier of oxygen in the lattice of Pr_2_NiO_4_ and Pr_2_Ni_5/6_Mo_1/6_O_4_ [186]. **d** Correlations of perovskite catalytic performance with oxygen p-band bulk descriptor [187]. **e** Theoretical ORR volcano plot. **f** Calculated Δ*G*_1_ (H_2_O(l) + * → HO* + H^+^  + e^−^), Δ*G*_2_ (HO* → O* + H^+^  + e^−^), Δ*G*_3_ (O* + H_2_O(l) → HOO* + H^+^  + e^−^), and Δ*G*_4_ (HOO* → O_2_(g) + H^+^  + e^−^) *vs.* Δ*G*(O*–HO*) [188]

Oxygen vacancy formation (ΔH_vacancy formation_) and migration energetics (ΔH_vacancy migration_) may be altered by strain, which is important for altering oxygen vacancy transport [180], oxygen surface exchange rate [181], and oxygen vacancy ordering [182]. The oxygen vacancy formation energy is reduced by moving the bulk O 2*p*-band center closer to the Fermi level, which improves the favorability of oxygen vacancy formation (Fig. 10b). The ease of electron transport from (to) oxygen states at the Fermi level upon oxygen addition (removal) and its connection with the weak metal–oxygen bond strength in bulk are the causes for this drop in vacancy formation energy [183]. Strain modifies the formation energy of oxygen vacancies in comparison with the unstrained bulk state via altering the O 2*p*-band center. By means of this strain-defect connection, the structural effects of strain are linked to the chemical ionic properties of perovskite oxides. These impacts from strain on oxygen vacancy and interstitial formation energies of perovskite oxides might affect the oxygen interstitial dissuasion, especially in Ruddlesden-Popper oxide family [184, 185]. The barriers to oxygen migration, which are frequently mediated by vacancies in perovskites, are also altered by strain. In La_2−x_Sr_x_NiO_4_, tensile strain has been found to reduce the barriers to interstitial migration of both oxide and peroxide interstitial species (Fig. 11a) [184]. Meyer et al. discovered that oxygen nonstoichiometry in strained cuprates is mediated by strain-modified surface exchange kinetics. Tensile-strained La_1.85_Sr_0.15_CuO_4_ (LSCO) exhibits an oxygen exchange rate that is about one order of magnitude faster than a compressively strained film (Fig. 11b). The reduced oxygen interstitial migration barrier can also be applied for oxygen transport in membranes. In these membranes, oxygen transport requires the coordination of several different processes, including membrane surface reactions for oxygen exchange from the molecular state to lattice oxygen and vice versa (oxygen reduction and evolution), as well as concurrent oxygen ionic and electronic conduction. For instance, Pr_2_Ni_1−x_Mo_x_O_4_ displays excellent oxygen transport performance due to a high interstitial oxygen mobility, with significantly reduced diffusion barrier of oxygen (Fig. 11c) [186]. Moreover, the adsorption energy of oxygen and oxygenated adsorbates (*OH, *OOH) on LaBO_3_ perovskites have been observed to change linearly with the O 2*p*-band center, making it an excellent electronic structural descriptor for surface reactivity during OER (Fig. 11d), and ORR (Fig. 11e) [187, 188]. The adsorption free energy of OER/ORR reaction intermediates at the bare surfaces may be used to generate theoretical OER/ORR volcano plots when the surfaces exhibit little coverage dependence. Both the ORR and OER activity volcano plots may be represented in the same volcano map by following the four OER/ORR charge transferring phases and making use of the scaling connections between surface reaction free energies and a surface binding energy descriptor. G(O*)-G(HO*) was used as the OER activity descriptor for the hypothetical OER activity volcano plot, and G(HO*) was used as the ORR activity descriptor for the hypothetical ORR activity volcano plot in order to more clearly show the trend (Fig. 11f) [188]. Because of the influence of strain on both bulk and surface energetics stated above, the capacity to controllably modify electrical and chemical properties has a substantial impact on not only room-temperature oxygen electrocatalysis but also high-temperature oxygen electrocatalysis.

**Fig. 12:**
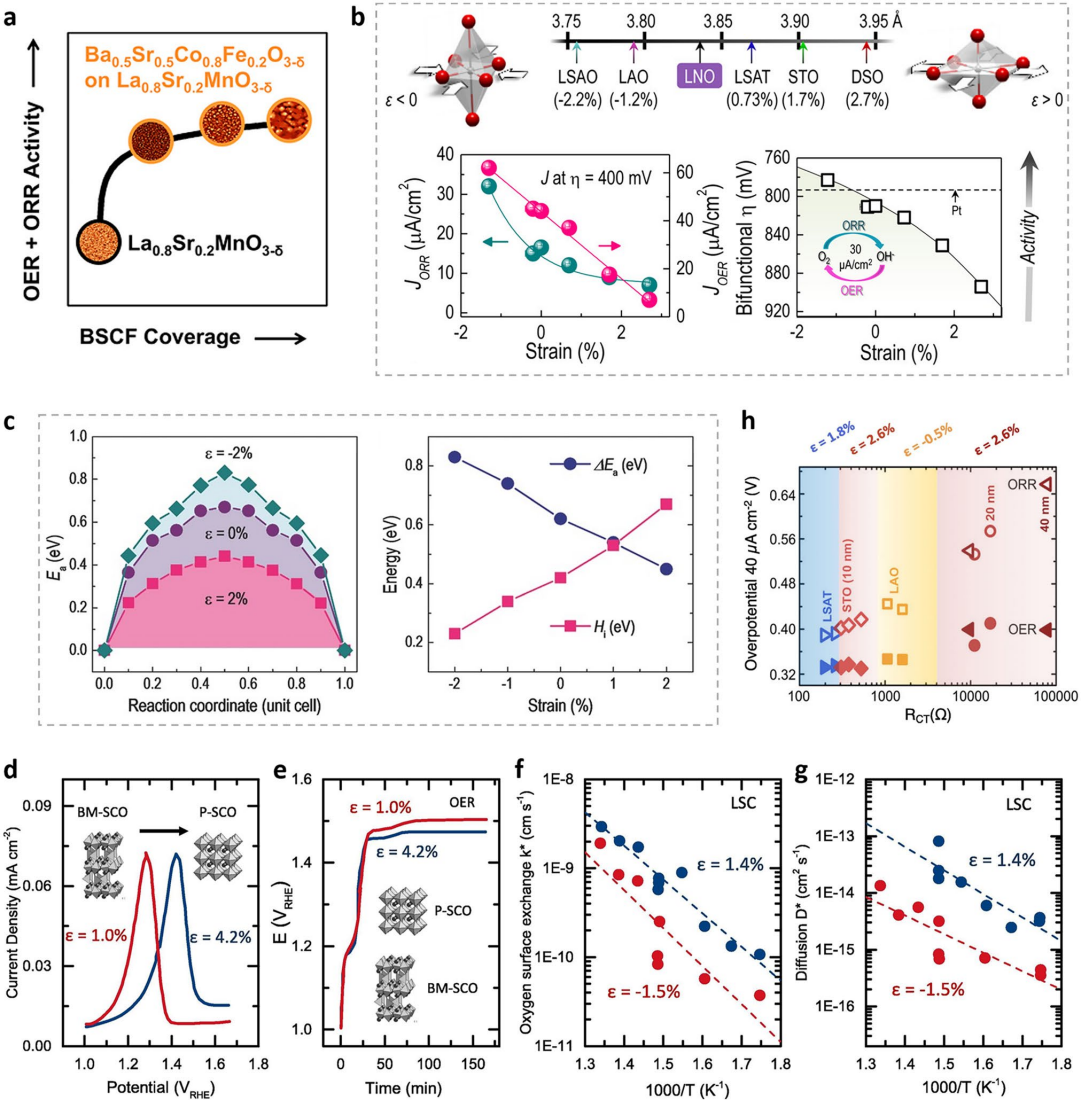
**a** Bifunctional composite catalyst for oxygen electrocatalysis (ORR and OER) [189]. **b** Enhanced ORR and OER bifunctional activities using compressive strain [110]. **c** Strain-dependent oxygen activation [190]. **d** Anodic scan around the topotactic transformation peak as brownmillerite SrCoO_2.5_ (BM-SCO) is oxidized to the perovskite SrCoO_3_ (P-SCO) at 10 mV s^−1^ scan rate [191]. **e** Galvanostatic stability scans at 5 μA under different tensile strains in O2-saturated 0.1 M KOH [191]. **f** k* and **g** D* coefficients as a function of temperature for La_0.8_Sr_0.2_CoO_3–δ_ (LSC) [181]. **h** The overpotential of OER (filled) and ORR (open) on LCO corresponds with the charge transfer resistance, R_CT_, measured at open circuit under different tensile strains (*ε* = 1.8%, 2.6%, and − 0.5%) [111]

The kind of proton–electron transfer that occurs during elementary reaction steps can be altered by the manipulation of electronic structure via strain effects [172]. The formation of thin films of Ba_0.5_Sr_0.5_Co_0.8_Fe_0.2_O_3−δ_ on La_0.8_Sr_0.2_MnO_3−δ_ improves the OER/ORR performance (Fig. 12a) [189]. Petrie et al. stretched the conducting perovskite LaNiO_3_ epitaxially to investigate its effect on ORR/OER (Fig. 12b) [110]. This work indicated that compressive strain considerably improves both processes, resulting in a bifunctional catalyst that outperforms precious metals. The enhanced bifunctionality is attributed to strain-induced splitting of the e_g_ orbitals, filling of the out-of-plane d_z2_ orbitals and altering the perovskite BO_6_ octahedral, modifying orbital asymmetry at the surface [110]. The addition of tensile strain to catalysts (e.g., cobaltites and nickelates) to increase oxygen vacancy formation has shown to be beneficial for the construction of higher activity catalysts. When a small biaxial tensile strain (2%) is applied to SrCoO_3_ films, the favorability for oxygen vacancy formation (with a 30% decrease in oxygen activation energy barrier) manifests itself as a minor increase in Co^3+^ and a decrease in Co^4+^ species (Fig. 12c) [190]. These results are further supported by the anodic oxidation studies of SrCoO_3_ films, where the film with the highest tensile-stress displayed a larger anodic potential for oxygen intercalation (Fig. 12d) as a result of increased tensile strain and a higher activity for OER (Fig. 12e) after the vacancy filling via oxygen intercalation [191]. The fact that tensile strain promotes the oxygen exchange kinetics and even oxygen transport was also observed in La_0.8_Sr_0.2_CoO_3−δ_ (LSC) (Fig. 12f, g) [181]. Additionally, strain can change the rate at which charges move across the interface between electrode and electrolyte. It is the origin of enhanced charge transfer process that led to higher OER catalytic current or lower overpotential for the OER/ORR happing. Meanwhile, strain is not a single isolated factor; the strain changes are always along with the lattice changes of crystal. Then, the metal–oxygen bond length, in another word, the covalent between metal and oxygen would also be adjusted. The adjusted oxidation state of metal sites would lead to modulated charge transfer rate and even catalytic activity. Thus, strain would affect the OER/ORR performance via affect the charge transfer rate. As shown in Fig. 12h, according to substrate-induced and film thickness-induced stresses in LaCoO_3_, moderately tensile-strained LaCoO_3_ thin films grown on LSAT (1.8% strain), grown on STO (2.6% strain), and finally compressively strained films grown on LAO (−0.5% strain), demonstrated the highest OER and ORR activity (LCO) [111]. To date, a great deal of research has been done on strain engineering to help with the design of oxygen electrocatalysts. However, it should be noted that properly characterizing and modulating the strain effect remains a very difficult task.

## Oxygen Electrocatalysis Related Applications

**Fig. 13:**
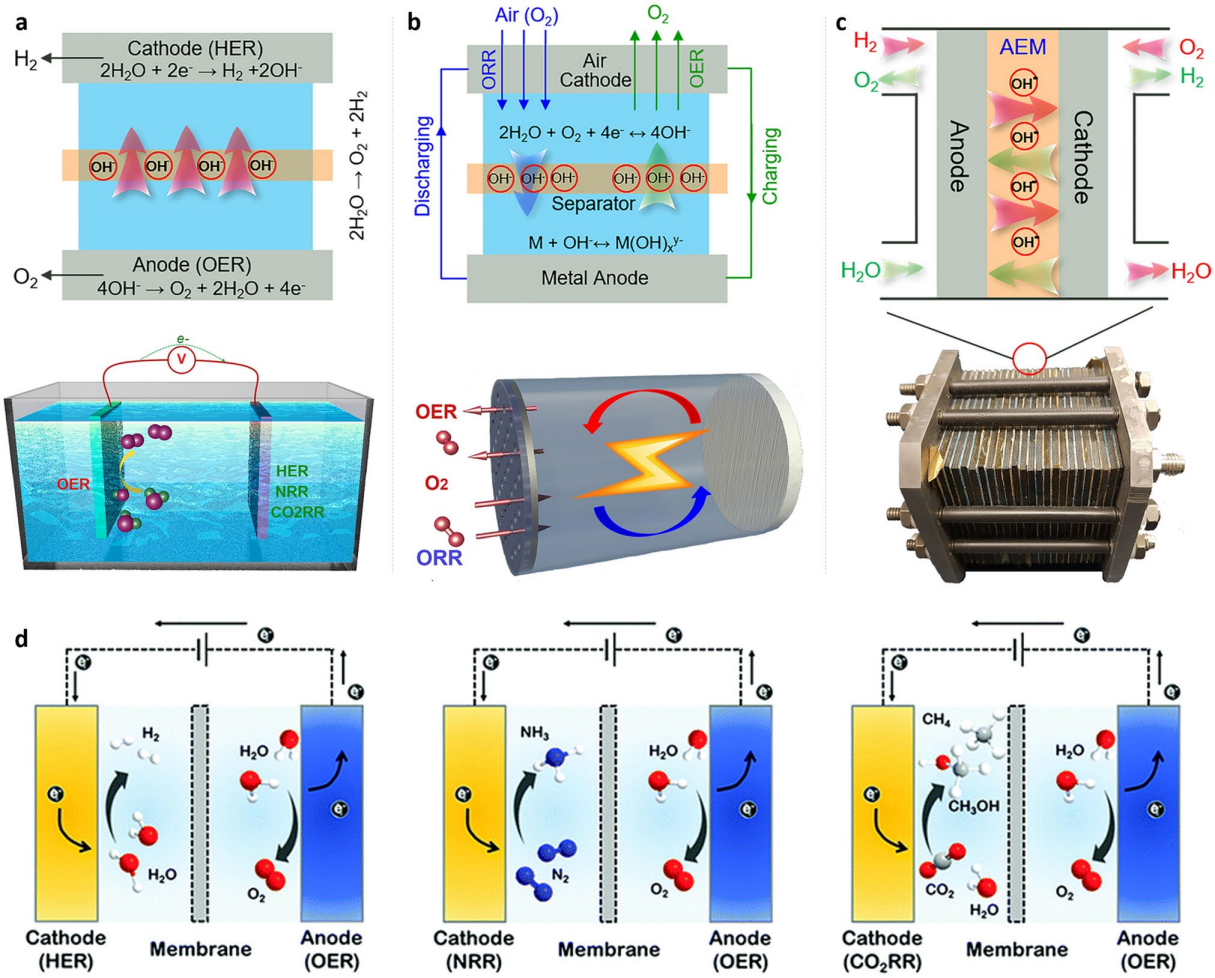
OER in electrochemical energy conversion and storage devices. **a** Electrolyzers for small molecules’ electrolysis. **b** Metal–air batteries. **c** Regenerative fuel cells [193]. **d** Scheme of several energy conversion electrolyzers relying on PEM: water electrolysis, nitrogen reduction and CO_2_ reduction

Exploration of high-performance energy conversion and storage (ECS) technologies such as regenerative fuel cells, metal–air batteries, and small molecule (N_2_, CO_2_, H_2_O) electrolyzers capable of harvesting, converting, and storing renewable energy in chemicals and then reconverting at the point of need is critical, but it remains a scientific challenge [192]. Because it supplies electrons in electrochemical reactions that convert (renewable) electrical energy and chemical fuels, the OER and/or ORR module is a critical component in modern ECS systems. Firstly, the OER happens at the anode of an electrolyzer used to generate chemical fuel by electrolysis of water molecules (Fig. 13a) [193]. For example, water splitting: oxygen electrocatalysts can also be used in water splitting applications, where water is split into oxygen and hydrogen [129, 194–196]. This process is used in the production of hydrogen fuel for use in fuel cells. Secondly, oxygen electrocatalysis is also used in metal–air batteries, where oxygen is the cathode reactant. Metal–air batteries have a high energy density and are being developed as a promising alternative to traditional lithium-ion batteries. In which the ORR occurs on the positive electrode in metal–air batteries depicted in Fig. 13b [193], its activity and stability impact the charging and discharging performances of metal–air batteries [130, 197, 198]. Among various oxide catalysts, the perovskite catalysts display excellent performance as bifunctional OER/ORR catalysts with potential for Zn-air battery (Table 3). Thirdly, oxygen electrocatalysis is a crucial aspect of fuel cell technology. In a fuel cell, oxygen is reduced at the cathode, producing water and generating electricity. A regenerative fuel cell, which functions in two modes of hydrogen production (electrolyzer cell mode) and power production (fuel cell mode), can only provide efficient long-term energy storage and on-demand conversion back to electrical energy through oxygen electrocatalysts (Fig. 13c) [193]. Oxygen electrocatalysts play a critical role in facilitating this process, improving the efficiency and performance of fuel cells. Acidic OER is more applicable compared to alkaline OER to commercialization because of the successful development and large-scale applications of proton exchange membranes (PEM), which have high proton conductivity, high voltage efficiency, low Ohmic loss, high current density, and high gas purity [55, 199]. More significant, an inherent advantage of acidic electrolytes over alkaline electrolytes is that hydronium ions have a far greater conductivity (350 S cm^2^ mol^−1^) than hydroxide ions (198 S cm^2^ mol^−1^). Thus, three energy conversion electrolyzers using PEM technology are shown in Fig. 13d, such as water electrolysis, nitrogen reduction and CO_2_ reduction. Meanwhile, ORR is mainly connected to metal–air batteries (Fig. 13b) [200] and regenerative fuel cells (Fig. 13d) [103]. Overall, oxygen electrocatalysis is a key area of research with numerous applications in clean energy production, environmental remediation and chemical synthesis.

**Table 3:** Summary of OER and ORR bifunctional oxygen perovskite electrocatalysts in 0.1 M KOH solution

Catalyst	E_onset_ ORR	E_onset_ OER	Tafel slope OER (mV dec^−1^)	Tafel slope ORR (mV dec^−1^)	Refs
La_0.8_Sr_0.2_MnO_3_	− 0.2 V vs, Ag/AgCl	0.60 V vs. Ag/AgCl			[201]
LaNi_0.85_Mg_0.15_O_3_		0.82	95	105	[202]
La_0.9_Sr_0.1_CoO_3_	0.806 V vs. RHE	1.612 V vs. RHE			[203]
Ba_0.9_Co_0.5_Fe_0.4_Nb_0.1_O_3−*δ*_	− 0.2 V vs, Ag/AgCl	0.59 V vs. Ag/AgCl			[204]
BaMnO_3_	− 0.19 V vs. Ag/AgCl	0.60 V vs. Ag/AgCl			[205]
LaCo_0.8_Mn_0.2_O_3_	− 0.21 V vs. Ag/AgCl	0.64 V vs. Ag/AgCl			[206]
Ba_0.5_Sr_0.5_Co_0.8_Fe_0.2_O_3_	− 0.18 V vs. Ag/AgCl	0.50 V vs. Ag/AgCl			[207]
BaTiO_3_	0.88 V vs. RHE	1.3 V vs. RHE			[208]
LaMnO_3_	0.78 V vs. RHE	154 mV dec^−1^		110.2 mV dec^−1^	[202]
(La_0.8_Sr_0.2_)_0.95_Mn_0.95_Ir_0.05_O_3_	− 0.05 V vs. Ag/AgCl	0.48 V vs. Ag/AgCl	103 mV dec^−1^		[209]
(La_0.8_Sr_0.2_)_0.9_Mn_0.9_Co_0.1_O_3_	− 0.09 V vs. Ag/AgCl	0.67 V vs. Ag/AgCl			[210]
LaCo_0.5_Ni_0.5_O_3_			73.9 mV dec^−1^	112 mV dec^−1^	[211]
La_0.95_FeO_3_	0.58 vs RHE	1.64 vs. RHE			[148]
La_0.6_Sr_0.4_CoO_3-δ_	− 0.187 vs Hg/HgO	0.893 vs. Hg/HgO			[212]
La_0.5_Sr_0.5_CoO_2.91_	0.78 vs RHE	1.84 vs. RHE			[213]

Oxygen electrocatalysis (OER and ORR) in solution is closely related to OER and ORR in solid oxide fuel cells (SOFC) and solid oxide electrolysis cells (SOEC). In addition to the oxygen electrocatalysis in aqueous system, oxide catalysts are also widely applied in SOFC and SOEC [214, 215]. There is a close relationship between OER and ORR, and SOFC and SOEC. These four processes all involve the electrochemical reactions of oxygen molecules. SOFC and SOEC are efficient energy conversion and storage systems that enable high-efficiency energy conversion, including electrochemical reactions where OER and ORR are important reactions [216, 217]. SOFC reacts hydrogen (or other fuels) and oxygen to produce electricity, water, and heat, where oxygen is reduced to oxygen ions. On the other hand, SOEC drives the electrochemical reduction of oxygen molecules to produce hydrogen or other combustible gases using electricity. Therefore, SOFC and SOEC are essentially the reverse processes of OER and ORR. In these processes, the activation energy of oxygen molecules can be adjusted by increasing the temperature or applying a voltage. By raising the temperature or applying a voltage, the energy of oxygen molecules will increase, which will help to reduce the activation energy of OER and ORR reactions, thereby improving reaction rates and efficiency. In addition, solid oxide materials serving as catalysts can also affect the rate and efficiency of OER and ORR reactions by adjusting their surface structures and electronic properties.

**Fig. 14:**
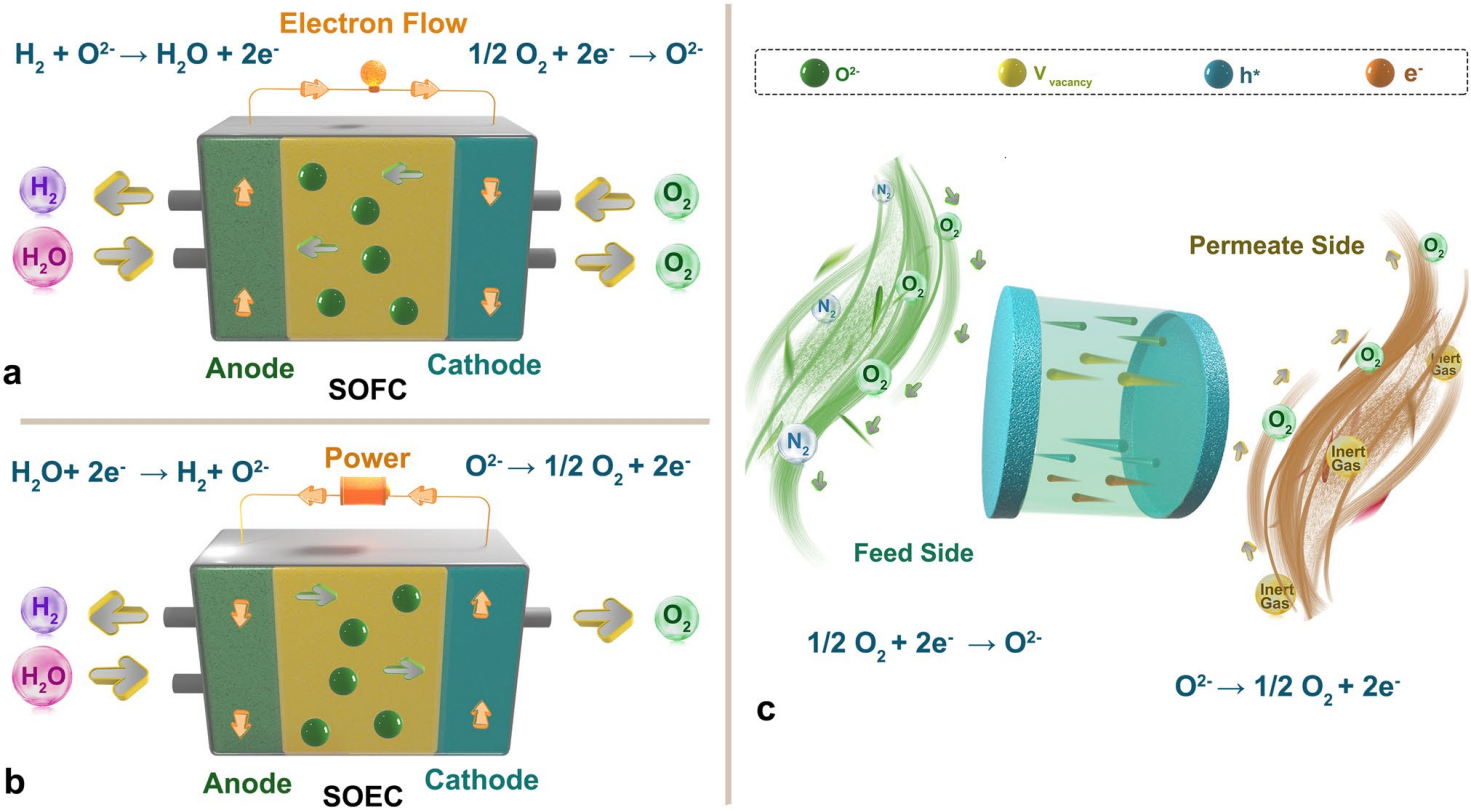
Schematic of working principle of **a** SOFC and **b** SOEC. **c** Schematic of working principle of MIEC materials-based oxygen separation membrane

A SOFC is an electrochemical device that can either generate electricity with high efficiency or operate in reverse mode (as electrolyzer, SOEC) to produce chemical compounds (fuels) from electricity (Fig. 14a, b). SOFC is a potential technology for assisting in decarbonization and the development of sustainable energy systems. The key benefits of this technology are the following. (1) Fuel Flexibility: as a SOFC may use hydrocarbon fuel as well as varied compositions of biofuel and hydrogen derived from renewable sources, this is essential to the zero-emission transition. (2) Efficiency: the performance of SOFC fuel cells is significantly higher than that of internal combustion and coal-fired engines. According to the 2019 Fuel Cell Industry Review, commercial SOFC electricity generators now have an efficiency of 55% to 60% to more than 80% when utilized in Combined Heat Power systems (CHP). (3) Water generation: SOFC generates water as a by-product of power generation, an obvious advantage over traditional energy sources. Fuel cell utilization in the energy industry has the potential to minimize water use and withdrawals. (4) Scalability: this is an imperative feature that may be provided by employing a distributed energy generator since the initial installation and operation do not necessitate significant capital expenditures and it can be expanded as demand increases. This is feasible because more fuel cell modules may be added in parallel to improve the power output. (5) Low carbon emission: SOFCs are regarded near-zero emission devices due to their operating principle. Furthermore, if the fuel is pure hydrogen, SOFC products are nothing more than water and heat. (6) Materials: the layers of the SOFC are constructed of ceramic, which eliminates the use of precious metal as catalytic material, making the device much cheaper.

To achieve good SOFC performance, the catalysts at the anode and the cathode are very important, while the electrolyte should have low electronic conductivity but high oxygen mobility [218–220]. At the anode, fuels such as H_2_ and natural gas are oxidized. Due to the high temperature inside the cell, anodic materials need to have high electrical conductivity, high stability, thermal compatibility with other cell components, electrocatalytic activity for oxidation reactions, and porosity for effective transport of carrier gases in a reducing atmosphere. Pure porous metallic electrodes, such as Ni, Pt and Ru, have been utilized as anodes [221]. A lot of perovskite-like oxides such as Ba_0.5_La_0.5_Ti_0.3_Mn_0.7_O_3_, Ba_0.5_La_0.5_In_0.3_Ti_0.1_Mn_0.6_O_3_ [222], and La_1−x_Sr_x_MnO_3_ [223] were also investigated as anode catalyst for SOFC. The reduction of oxygen to O^2−^ ions on the surface into lattice vacancies is required for catalysts at cathode. Oxides such as cobalt perovskites (LaCoO_3_, SrCoO_3_, and La_1−x_Sr_x_CoO_3−δ_), iron-contained perovskites (SrFeO_3_ and La_1−x_Sr_x_FeO_3−δ_), and Ni/Cu-layered perovskite-like oxides [224–227] with good mixed oxygen ionic and electronic conductivity (MIEC) are widely applied as cathode in SOFC [228].

In SOFC, OER and ORR occur at the cathode and anode, respectively, driving the fuel cell reaction to generate electricity. In SOEC, OER and ORR also occur at the anode and cathode, enabling water splitting and hydrogen production through reverse current. However, the solid oxide electrolyte used in SOFC and SOEC requires high temperature to operate, which poses many technical challenges in practical applications, such as thermal stability and mechanical strength. In contrast, water-based OER and ORR typically occur at lower temperatures, making them more suitable for low-temperature energy conversion and storage applications. By studying the catalytic mechanism and catalyst design of OER and ORR, more efficient and stable catalysts can be provided for solid SOFC and SOEC, enabling more sustainable energy conversion and storage. Moreover, based on the intrinsic oxygen reduction property as cathode in SOFC and oxygen evolution property as anode in SOEC with, these catalysts are also widely investigated directly as oxygen transport membrane [229, 230]. Oxygen has been recognized as an essential chemical for power plants based on fossil fuel consumptions such as integrated gasification combined cycle and oxy-fuel combustion, which have been recognized as the most realistic clean energy technologies for energy production with carbon capture and storage. Unfortunately, most of the current industrial oxygen production is achieved by pressure swing adsorption and the cryogenic distillation process. These conventional methods are costly and energy-intensive, releasing substantial amounts of CO_2_ release. Dense ceramic membranes made from MIEC materials have attracted interest due to their advantages such as high selectivity (nearly 100% for oxygen) and energy- and cost-efficiency [231–233]. Among the MIEC materials studied, perovskite oxides are the focus of research due to their high oxygen permeation flux rates [186, 234–236] For such kinds of membranes, the oxygen transport requires a good match of multiple functions: membrane surface reactions for oxygen exchange from the molecular state to lattice oxygen (oxygen reduction) and vice versa (oxygen evolution); and the concomitant ionic oxygen and electronic conduction (Fig. 14c) [237].

## Conclusion and Outlook

In conclusion, the rational design of oxide catalysts for oxygen electrocatalysis has the potential to revolutionize various fields, from clean energy production to and chemical synthesis. By understanding the fundamental principles underlying the catalytic activity of oxides, researchers can design new catalysts with enhanced performance and durability [238–241]. To designing oxide catalysts is to manipulate their electronic and geometric structures. This can be achieved through the use of element doping, defecting engineering, among other techniques. These modifications can enhance the catalytic activity of oxides by creating active sites, increasing the surface area, and optimizing the electronic properties of the catalyst. Moreover, to explore new oxide materials with unique properties, such as perovskites, spinels, and layered double hydroxides. These materials offer advantages such as high conductivity, high surface area, and structural diversity, which can be leveraged to enhance the catalytic activity of the oxide. OER and ORR have attracted considerable attention due to their potential use in various energy conversion and storage systems, including water electrolyzers, metal–air batteries, electrochemical energy conversion and storage systems. However, despite significant progress made in the design of oxide catalysts for OER and ORR, there are still several challenges that need to be addressed to enable their widespread use.

- To improve the catalytic activity of oxide catalysts towards ORR or OER. Although many oxide catalysts have been reported to exhibit good ORR or OER activity, their performance still falls short of that of precious metal-based catalysts. This is partly due to the sluggish kinetics and complex reaction mechanisms of ORR or OER, which require highly active and selective catalysts. Therefore, there is a need for further research into the fundamental mechanisms underlying the ORR or OER process, and how they can be tailored to enhance the activity of oxide catalysts.
- To improve the stability of oxide catalysts under the harsh operating conditions of electrochemical devices. Many oxide catalysts are prone to degradation and deactivation due to the corrosive nature of the electrolyte, high temperature, and high pressure. Moreover, the formation of oxide layers and surface defects can also lead to the loss of catalytic activity over time. Therefore, there is a need for the development of new synthesis and processing methods to improve the stability and durability of oxide catalysts under such conditions.
- To improve the scalability and cost-effectiveness of the synthesis of oxide catalysts. Most oxide catalysts are synthesized through high-temperature solid-state reactions or sol–gel methods, which are often time-consuming, energy-intensive, and difficult to scale up. Therefore, there is a need for the development of new synthesis methods that can produce oxide catalysts at low cost and in large quantities, while maintaining their high activity and stability.
- To deeply explore the reaction mechanism of oxide catalysts during oxygen electrocatalysis. Investigate the real active sites/centers and the mechanism, isotope tracing experiments should be conducted. For example, the oxide catalysts for OER will happen via two different reaction mechanisms: adsorbate evolution mechanism (AEM) and lattice oxygen-mediated mechanism or lattice oxygen oxidation mechanism (LOM). The real reaction process/mechanism of the synthesized catalyst could be investigated via ^18^O isotope detection of the reaction product as well as density functional theory calculations. It will directly detect the participation of oxygen anions from the oxide lattice as an active intermediary in the OER.
- To explore the intermediates will give rise to the understanding of the reaction process/step and the real mechanism. Thus, it is suggested to further explore the intermediates combined with the isotope tracing experiments, theoretical calculations, and direct proof via in situ characterizations. For example, in situ attenuated total reflection surface-enhanced infrared absorption spectroscopy (ATR-SEIRAS) measurement was highly sensitive to oxygen-containing intermediates, which could be introduced into OER and ORR investigation. More in situ characterization experiments should be conducted for the deep understanding of the real relationship between catalysts and catalytic performance (stability and activity) from phase structure to oxidation state. The in situ X-ray absorption spectroscopy (XAS) measurement could be introduced as in situ X-ray absorption near edge structure (XANES) is useful for in situ oxidation state exploration. The in situ extended X-ray absorption fine structure (EXAFS) is useful for the in situ crystal exploration. This directly obtained information can help to understand the reaction mechanisms and thus guide the design of promising catalysts.

Oxide catalysts hold great promise for use in electrochemical energy conversion and storage systems, but there are significant challenges that need to be addressed in their development for oxygen reduction and oxygen evolution reactions. Improving their catalytic activity, stability, and scalability is crucial for their widespread adoption and for reducing our reliance on fossil fuels, while also mitigating the environmental impact of energy production. The rational design of oxide catalysts for oxygen electrocatalysis is critical for advancing the development of fuel cells, metal–air batteries, water splitting, and carbon capture technologies, which can significantly reduce greenhouse gas emissions, improve energy efficiency, and enhance the sustainability of our energy systems. Continued research in this area is of utmost importance for a cleaner and more sustainable future. By addressing the challenges associated with oxide catalysts for ORR and OER, we can make significant strides towards a cleaner and more sustainable future. The development of these catalysts has the potential to revolutionize the way we produce and store energy, ultimately making our energy systems more efficient and environmentally friendly. Therefore, continued investment in research in this area is essential to unlock the full potential of oxide catalysts and their applications.
